# Cytoplasmic innate immune sensing by the caspase-4 non-canonical inflammasome promotes cellular senescence

**DOI:** 10.1038/s41418-021-00917-6

**Published:** 2021-12-16

**Authors:** Irene Fernández-Duran, Andrea Quintanilla, Núria Tarrats, Jodie Birch, Priya Hari, Fraser R. Millar, Anthony B. Lagnado, Vanessa Smer-Barreto, Morwenna Muir, Valerie G. Brunton, João F. Passos, Juan Carlos Acosta

**Affiliations:** 1 Cancer Research UK Edinburgh Centre, MRC Institute of Genetics and Cancer, University of Edinburgh, Crewe Road, Edinburgh, EH4 2XR UK; 2 MRC London Institute of Medical Sciences, Hammersmith Hospital Campus, Du Cane Road, London, W12 0NN UK; 3 Robert and Arlene Kogod Center on Aging, Mayo Clinic, Rochester, MN 55905 USA; 4 Instituto de Biomedicina y Biotecnología de Cantabria, IBBTEC (CSIC, Universidad de Cantabria). C/ Albert Einstein 22, Santander, 39011 Spain

**Keywords:** Cell biology, Proteolysis, Immune cell death

## Abstract

Cytoplasmic recognition of microbial lipopolysaccharides (LPS) in human cells is elicited by the caspase-4 and caspase-5 noncanonical inflammasomes, which induce a form of inflammatory cell death termed pyroptosis. Here we show that LPS-mediated activation of caspase-4 also induces a stress response promoting cellular senescence, which is dependent on the caspase-4 substrate gasdermin-D and the tumor suppressor p53. Furthermore, we found that the caspase-4 noncanonical inflammasome is induced and assembled in response to oncogenic RAS signaling during oncogene-induced senescence (OIS). Moreover, targeting caspase-4 expression in OIS showed its critical role in the senescence-associated secretory phenotype and the cell cycle arrest induced in cellular senescence. Finally, we observed that caspase-4 induction occurs in vivo in mouse models of tumor suppression and ageing. Altogether, we are showing that cellular senescence is induced by cytoplasmic LPS recognition by the noncanonical inflammasome and that this pathway is conserved in the cellular response to oncogenic stress.

## Introduction

Cellular senescence is a cell state characterized by a proliferative cellular arrest, a secretory phenotype, macromolecular damage, and altered metabolism that can be triggered by several different stress mechanisms [1]. Senescent cells produce and secrete a myriad of soluble and insoluble factors, including cytokines, chemokines, proteases, and growth factors, collectively known as the senescence-associated secretory phenotype (SASP) [2–4] and interleukin-1 (IL-1) signaling is one of its critical signaling pathways [5, 6]. The role of the SASP in cancer is complex and mechanistically ill-defined in ageing-associated diseases, two of the critical pathophysiological contexts where senescence is functionally relevant [7, 8]. More recent evidence proposes that different triggers might induce distinctive SASP subsets with concrete functions [9]. Nonetheless, the SASP has started to incite interest as a potential therapeutic target in disease [10, 11]. Therefore, a better understanding of the molecular machinery regulating the SASP is needed.

Pattern recognition receptors (PRRs) of the innate immune system are molecular sensors that are activated by microbial-derived pathogen-associated molecular patterns (PAMPs) or by damage-associated molecular patterns (DAMPs or alarmins) generated endogenously in cells under certain conditions of stress and damage [12]. Emerging data indicate a close relationship between these PRRs and cellular senescence. For example, the SASP is controlled in oncogene-induced senescence (OIS) by serum amyloid signaling through the PRR toll-like receptor-2 (TLR2), downstream nucleic acid sensing by the cGAS-STING pathway [13–15]. Moreover, we have previously shown that inflammasomes are critical for the SASP [5]. Inflammasomes are multiprotein platforms that induce the proteolytic activity of the inflammatory cysteine-aspartic protease caspase-1, which activates by proteolytic cleavage the proinflammatory cytokines IL-1β and interleukin-18 (IL-18). The canonical inflammasomes are assembled by PRRs of the nod-like receptor family, pyrin, or by the cytoplasmic DNA sensor AIM2 [16, 17]. Alternatively, the related inflammatory caspase-4 and caspase-5 (caspase-11 in mice) function as independent PRRs for cytoplasmic microbial lipopolysaccharide (LPS) activating a noncanonical inflammasome. Critically, activated noncanonical inflammasomes cleave the effector protein gasdermin-D, which induce a form of inflammatory programmed cell death termed pyroptosis [18–20]. Because the mechanism of SASP regulation by inflammasomes remains ill-defined, we decided to define the role of these inflammatory caspases in senescence. We show here that caspase-4 activation by cytoplasmic LPS triggers a senescence phenotype. Nonetheless, caspase-4 induction and noncanonical inflammasome assembly were observed in RAS^G12V^ mediated OIS. Moreover, we show here that the caspase-4 noncanonical inflammasome contributes critically to the establishment of the SASP and the reinforcement of the cell cycle arrest program during OIS, in a mechanism that is independent on its catalytic activity over its downstream pyroptotic target gasdermin-D. In all, we describe a new and critical function for cytoplasmic sensing by the caspase-4 noncanonical inflammasome in cellular senescence.

## Results

### Cytoplasmic LPS recognition by caspase-4 induces a senescent response in human diploid fibroblasts

While the canonical inflammasome can be activated with the microbial-derived molecule muramyl-dipeptide (MDP), the caspase-4 and caspase-5 noncanonical inflammasomes detect cytoplasmic bacterial LPS inducing gasdermin-D cleavage and pyroptosis. Gasdermin-D is the best characterized functional substrate of noncanonical inflammasomes, eliciting IL-1β secretion and pyroptotic cell death [19–21]. To compare the effect of activating the canonical and noncanonical inflammasome in senescence, we activated the caspase-1 or the caspase-4/5 inflammasomes by transfection of MDP or LPS in human IMR90 fibroblasts respectively. Similarly to other human cells expressing caspase-4 [18], IMR90 cells were sensitive to intracellular LPS in a dose-dependent manner (Figs. S1A, B, C). In contrast, MDP transfection did not induce cell death (Fig. S1A). LPS addition without further electroporation did not result in cell death, confirming the requirement of an intracellular location for LPS to trigger pyroptosis (Fig. S1C). Contrary to the myeloid cell line THP-1, we could not detect *CASP5* mRNA expression, confirming that its expression is mostly restricted myeloid cells (Fig. S1D). Thus, we focused on targeting caspase-4 and gasdermin-D but not caspase-5 to interrogate the effect of interfering with the noncanonical inflammasome in IMR90 cells in pyroptosis and senescence.

**Fig. 1:**
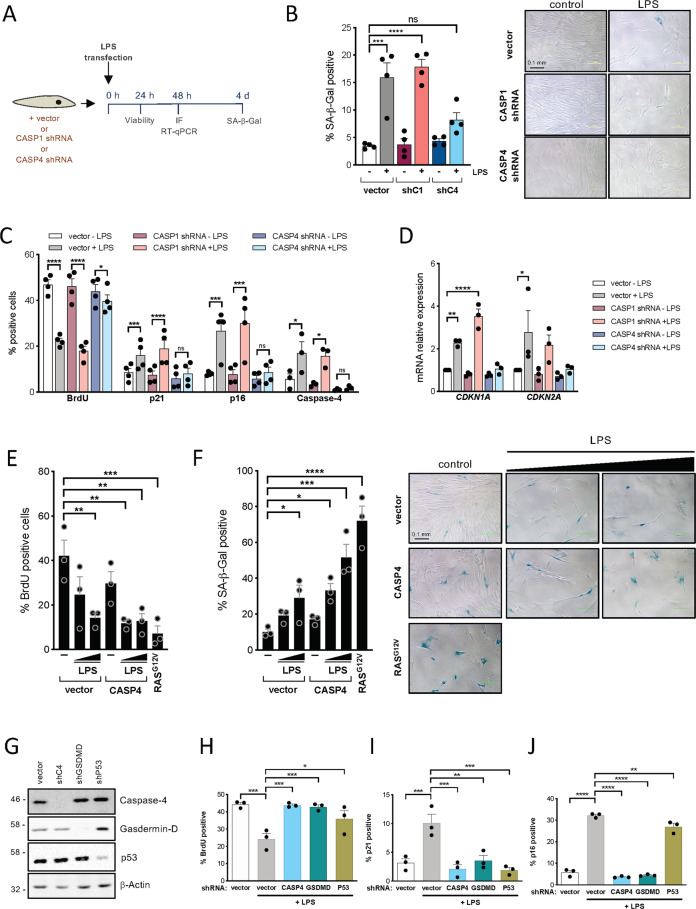
LPS-mediated caspase-4 activation induces a senescent phenotype in human primary fibroblasts. **A** Schematic representation of LPS transfection experiments shown in B-D and Supplementary Fig. 1 E–G. IMR90 cells were infected with an empty pGIPZ vector (vector) or shRNA targeting either *CASP1* (shC1) or *CASP4* (shC4) before transfection with 0.1 μg LPS. The acquisition of senescent features after LPS transfection was assessed by immunofluorescence of senescence markers, mRNA expression analysis by RT-qPCR and SA-β-Galactosidase activity. **B** SA-β-Galactosidase activity was determined in IMR90 cells 4 days after LPS transfection. Representative images for SA-β-Gal activity are shown. Graph bars, error bars, and dots represent respectively the mean ± standard error of the mean (s.e.m.) and the individual values of 4 independent experiments. Statistical analysis was performed using one-way analysis of variance (ANOVA). **C** BrdU incorporation and p16^INK4a^, p21^CIP1^, and caspase-4 protein expression levels were measured by immunofluorescence in IMR90 cells 48 h after LPS transfection. Graph bars, error bars and dots represent respectively the mean ± s.e.m. and the individual values of 4 independent experiments. Statistical analysis was performed using two-tailed Student’s *t* test. **D** *CDKN1A* (p21^CIP1^) and *CDKN2A* (p16^INK4a^) mRNA relative expression was quantified by RT-qPCR in IMR90 cells 48 h after transfection. Graph bars, error bars and dots represent respectively the mean ± s.e.m. and the individual values of 3 independent experiments. Statistical analysis was performed using one-way analysis of variance (ANOVA). **E** *CASP4* was overexpressed prior to LPS transfection in IMR90 cells. 5 ×105 control (vector) and CASP4 expressing IMR90 cells were transfected with increasing concentrations of LPS (0.1 or 1 μg), and cell proliferation was measured by BrdU incorporation 48 h after transfection. Graph bars, error bars, and dots represent respectively the mean ± s.e.m. and the individual values of 3 independent experiments. Statistical analysis was performed using one-way analysis of variance (ANOVA). **F** IMR90 cells were treated as in (**E**) and SA-β-Galactosidase activity was determined 4 days after LPS transfection. Representative images for SA-β-Galactosidase assay are shown. Graph bars, error bars, and dots represent respectively the mean ± s.e.m. and the individual values of 3 independent experiments. Statistical analysis was performed using one-way analysis of variance (ANOVA). **G** IMR90 cells were infected with an empty pGIPZ vector (vector) or shRNA targeting either *CASP4* (shC4), *GSDMD* (shGSDMD) or *TP53* (shP53) and protein expression analysis for Caspase-4, Gasdermin-D, p53, and β-Actin as loading control was performed by western blot. **H**–**J** IMR90 cells were transduced with an empty pGIPZ vector (vector) or shRNAs targeting either *CASP4* (shC4), *GSDMD* (shGSDMD) or *TP53* (shP53) prior to transfection with 0.1 μg LPS. BrdU incorporation (**H**) and the levels of p21^CIP1^ (**I**) and p16^INK4a^ (**J**) were measured by immunofluorescence in IMR90 cells 48 h after LPS transfection. Graph bars, error bars and dots represent respectively the mean ± s.e.m. and the individual values of 3 independent experiments. Statistical analysis was performed using one-way analysis of variance (ANOVA). ****P < 0.0001, ***P < 0.001, **P < 0.01, and *P < 0.05. ns, not significant. Scale bar = 0.1 mm as indicated.

Knockdown of caspase-4 and gasdermin-D, but not caspase-1 impaired cell death mediated by LPS transfection, confirming that the cells were dying by pyroptosis (Fig. 1G and Figs. S1E, F, G, H, I). Noticeably, cell death was detectable within the first hours after LPS transfection (Fig. S1J). However, the fraction of cells surviving cell death after these hours remained stable and viable (Fig. S1K), and this subpopulation was further examined.

Interestingly, this subset of cells displayed features of senescent cells such as decreased cell proliferation, increased levels of the cyclin-dependent kinase inhibitors p16^INK4a^ and p21^CIP1^ and increased senescence-associated-β-Galactosidase (SA-β-Galactosidase) activity (Fig. S1L, M). In contrast, MDP transfection did not induce a senescent response (Fig. S1M), indicating a specific role for the noncanonical inflammasome in PAMP-induced cellular senescence. Furthermore, the acquisition of a senescent phenotype was also accompanied by increased levels of caspase-4 (Fig. S1M).

To investigate if cell death debris or intracellular content released during pyroptosis contribute to the observed senescence induction by LPS, we investigated the effect of transferring pyroptotic cell culture supernatants in normal cells. We transfected IMR90 cells with 5 ug/mL of LPS and MDP as a control to produce a robust pyroptotic response. In such conditions, LPS induced a substantial decrease in cell viability (Fig. S2A). Twenty-four hours after LPS transfection, we transferred the supernatants of pyroptotic and control cultures into healthy IMR90 to assess the effect on cell proliferation and SA-β-Galactosidase activity (Fig. S2B, C, D). While LPS transfection induced a strong senescent response, shown by reduced cell proliferation and significant induction of SA-β-Galactosidase activity, supernatants enriched in dead cells from those cultures did not induce senescent markers significantly (Fig. S2B, C, D), suggesting that pyroptotic cell death contributes marginally to LPS induced senescence.

To assess if cytoplasmic LPS also induces cellular senescence in cells other than IMR90 fibroblasts, we conducted LPS transfection experiments in epithelial cell lines of the pancreas (CAPAN-1 and PSN-1), lung (A549) and colon (HCT116), observing that LPS transfection decreased the cell proliferation and induced SA-β-Galactosidase in all cases (Fig. S2E, F, G), suggesting that LPS transfection induces cellular senescence in other cell types from epithelial origin.

To examine the contribution of inflammatory caspases to the acquisition of a senescent phenotype following LPS transfection, we downregulated the expression of *CASP1* or *CASP4* with shRNA before transfection (Fig. 1A). In contrast to caspase-1, caspase-4 was required for the acquisition of senescent features, such as increased SA-β-Galactosidase activity, decreased cell proliferation, and induction of p21^CIP1^ and p16^INK4a^ in the subpopulation of cells surviving cell death (Fig. 1B–D). Next, human *CASP1* and *CASP4* were ectopically expressed in IMR90 fibroblasts (Fig. S2H). Overexpression of *CASP1* or *CASP4* alone resulted in a mild senescence induction with reduced cell proliferation and increased SA-β-Galactosidase activity (Fig. S2I, J). However, *CASP4* overexpression exacerbated the acquisition of senescent features in the presence of intracellular LPS (Fig. 1E, F and Fig. S2K), indicating that caspase-4 expression is critical for noncanonical inflammasome induced senescence. Overall, these results suggest that the acquisition of a senescent phenotype following intracellular LPS exposure is mediated through caspase-4 in a dose-dependent manner.

We decided to investigate the role of the caspase-4 substrate gasdermin-D and the critical senescence regulator p53 in LPS-induced senescence and pyroptosis by targeting their expression with shRNAs. In contrast to *GSDMD*, *TP53* knockdown failed to impair LPS-mediated cell death, indicating a negligent role for p53 regulating caspase-4 dependent pyroptosis (Fig. S1L). However, *TP53* and *GSDMD* knockdown significantly reduced LPS dependent cell growth arrest, and p53 and p21^CIP1^ induction (Fig. 1H, I, J and Fig. S2L). Interestingly, *GSDMD* knockdown also had a strong effect on p16^INK4a^ and caspase-4 induction during LPS-induced senescence (Fig. 1J, Fig. S2M). Altogether, these results indicate that cytoplasmic LPS sensing by the noncanonical inflammasome induced a senescence response that is dependent on caspase-4, gasdermin-D, and p53 expression.

### The caspase-4 mediated LPS-induced senescent response is independent of IL-1β priming

**Fig. 2:**
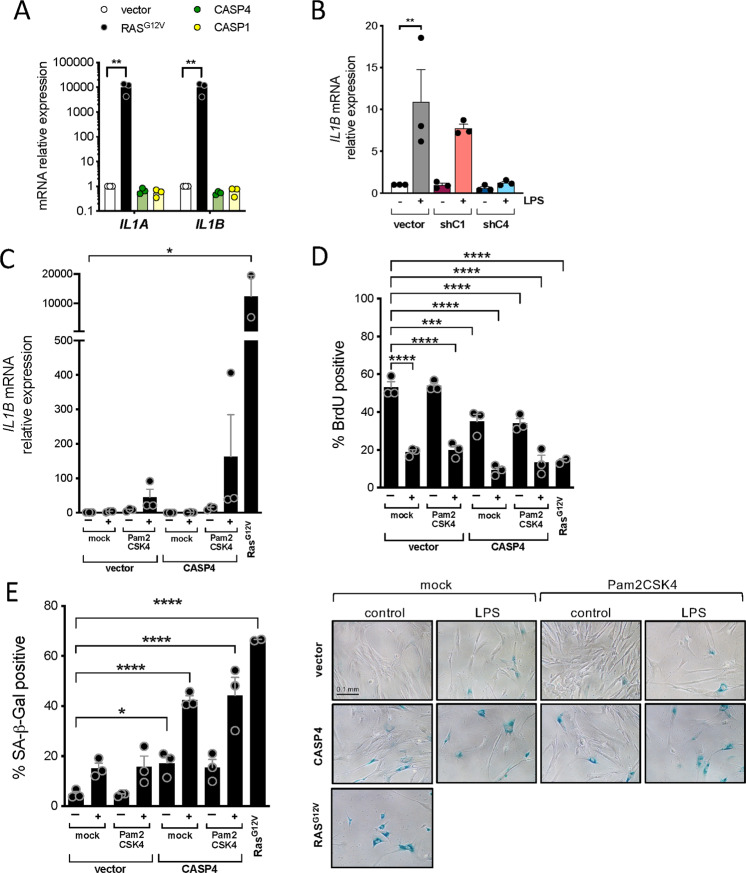
LPS-mediated caspase-4 induced senescence is independent of inflammasome priming. **A** *CASP4*, *CASP1* or *RAS*^G12V^ were overexpressed in IMR90 cells, *IL1A* and *IL1B* mRNA relative expression levels were quantified by RT-qPCR. Graph bars, error bars and dots represent respectively the mean ± s.e.m. and the individual values of 3 independent experiments. Statistical analysis was performed using one-way analysis of variance (ANOVA). **B** Cells were treated as shown in Fig. 1A. *CASP1* or *CASP4* expression was targeted by shRNA prior to LPS transfection with 0.1 μg LPS. *IL1B* mRNA relative expression was quantified by RT-qPCR 48 h after LPS transfection. Graph bars, error bars and dots represent respectively the mean ± s.e.m. and the individual values of 3 independent experiments. Statistical analysis was performed using one-way analysis of variance (ANOVA). **C**–**E** IMR90 cells were infected with *CASP4* or *RAS*^G12V^ expression vectors or empty vector (vector) control. After 3 h treatment with Pam2CSK4, cells were transfected with 0.1 μg LPS. *IL1B* mRNA relative expression (**C**) and BrdU incorporation (**D**) were measured by IF and RT-qPCR respectively 48 h after LPS transfection. **E** SA-β-Galactosidase activity was determined 4 days after LPS transfection. Representative images for SA-β-Galactosidase activity are shown. Graph bars, error bars and dots represent respectively the mean ± s.e.m. and the individual values of 3 independent experiments. Statistical analysis was performed using one-way analysis of variance (ANOVA). ****P < 0.0001, ***P < 0.001, **P < 0.01, and *P < 0.05. Scale bar = 0.1 mm as indicated.

Because overproduction of activated IL-1β can have detrimental effects [22], the inflammasome activation is tightly regulated by a two-step mechanism. In some cases, a first signal, also called priming, is required to boost transcriptional levels of *IL1B*. The priming signal is then followed by a second signal which induces the assembly of the inflammasome [16]. Intriguingly, senescence induction by the sole overexpression of *CASP4* or *CASP1* in IMR90 fibroblasts did not induce transcriptional activation of *IL1B* (Fig. 2A). In contrast, *IL1B* transcriptional levels were increased upon LPS transfection in a caspase-4 dependent fashion (Fig. 2B). We have previously shown that TLR2 has a role in controlling *IL1B* expression and the SASP in cellular senescence [14]. Thus, we decided to investigate if TLR2 mediated inflammasome priming could synergize with LPS-mediated caspase-4 induced senescence. As expected, the addition of the synthetic lipopeptides Pam2CSK4 and Pam3CSK4 (TLR2/6 and TLR1/2 agonists, respectively) but not LPS (TLR4 agonist) nor MDP to IMR90 cells highly induced *IL1B* mRNA levels (Fig. S3A). Then, we observed that priming the inflammasome with the TLR2 agonist Pam2CKS4 significantly synergizes with LPS transfection to produce a robust IL-1β induction, and this induction was further enhanced by *CASP4* ectopic overexpression (Fig. 2C). However, we did not observe a significant decrease in cell proliferation or SA-β-Galactosidase induction in LPS-induced senescence by the addition of Pam2CKS4 (Fig. 2D, E). Similar results were observed when TLR2 overexpressing cells were primed with the endogenous senescence-associated TLR2 alarmin A-SAA [14] or Pam2CKS4 with LPS transfection, which synergized in producing robust *IL1B* and SASP induction but without affecting dramatically LPS-induced proliferative arrest or SA-β-Galactosidase activity (Fig. S3, B, C, D, E, F). However, the observed increase in *IL1B* mRNA levels in LPS transfected cells in all conditions is minimal if compared to the logarithmic increase observed in OIS (Fig. 2C). These results suggest that additional signals to caspase-4 stimulation such as a sustained priming signaling are necessary for a robust *IL1B* and SASP induction during LPS-mediated cellular senescence. Moreover, these data suggest that the LPS-induced caspase-4 senescent response is independent of *IL1B* and the SASP.

### The caspase-4 proteolytic activity is not necessary for LPS-induced senescence

**Fig. 3:**
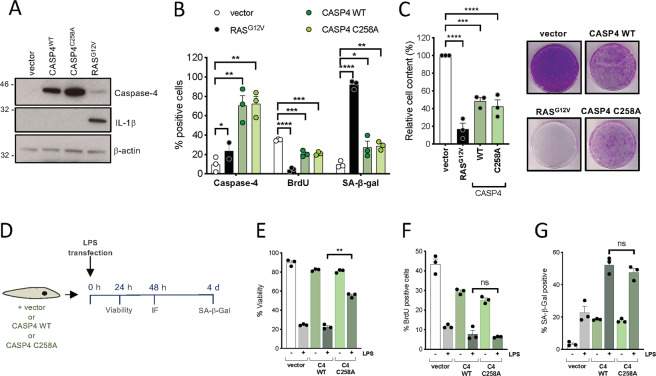
Caspase-4 mediated regulation of senescence is independent of its catalytical function. **A**–**C** IMR90 cells were infected with wild-type (WT) *CASP4*, catalytically inactive (C258A) *CASP4* or the empty vector (vector). Overexpression of *RAS*^G12V^ was used as a positive control for the induction of senescence. **A** Caspase-4 and IL-1β expression levels were investigated by immunoblotting. β-Actin immunoblot was performed for loading control. **B** Caspase-4 protein expression levels and BrdU incorporation measured by immunofluorescence, as well as SA-β-Galactosidase activity were assessed 4 days after equal number of cells were seeded. **C** Relative cell content (left) was quantified 15 days after equal number of cells were seeded; representative images (right) of crystal violet stained cells are shown. Graph bars, error bars and dots represent respectively the mean ± s.e.m. and the individual values of 3 independent experiments. Statistical analysis was performed using one-way analysis of variance (ANOVA). **D**–**G** (**D**) Schematic representation of the experiment shown in D-F and Supplementary Fig. 3G–H. IMR90 cells were infected with wild-type (WT) *CASP4*, catalytically inactive (C258A) *CASP4* or the empty vector (vector) prior to transfection with 1 μg LPS. (**E**) Cell viability was measured 24 h after LPS transfection, (**F**) BrdU incorporation was measured by immunofluorescence 48 h after LPS transfection and (**G**) SA-β-Galactosidase activity was determined 4 days after LPS transfection. Graph bars, error bars and dots represent respectively the mean ± s.e.m. and the individual values of 3 independent experiments. Statistical analysis was performed using two-tailed Student’s *t* test. ****P < 0.0001, ***P < 0.001, **P < 0.01, and *P < 0.05. ns, not significant.

The active site of human caspase-4 has been well characterized and is associated to the residue C258 [23], and point mutations of this amino acid renders the protein catalytically inactive [18, 24]. To further study the contribution of the protease activity of caspase-4 protease to senescence, *CASP4* wild-type and the catalytically death mutant C258A (*CASP4*^C258A^) were overexpressed in IMR90 cells (Fig. 3A), and the phenotypical outcomes were assessed. Overexpression of wild-type *CASP4* or *CASP4*^*C258A*^ reduced cell proliferation and increased SA-β-Galactosidase activity to a similar extent (Fig. 3B, C). Next, *CASP4* wild-type and *CASP4*^*C258A*^ were stably overexpressed in IMR90 fibroblasts before LPS transfection (Fig. 3D). *CASP4*^*C258A*^ but not *CASP4* wild-type expressing IMR90 cells were resistant to cell death after LPS transfection (Fig. 3E and Fig. S3G), indicating a dominant negative role for the caspase-4 defective form in pyroptosis. However, both *CASP4* wild-type and *CASP4*^*C258A*^ overexpressing cells remained equally sensitive to the acquisition of senescent features after LPS challenge (Fig. 3F, G and Fig. S3H). These results suggest that, in contrast to caspase-4 mediated pyroptosis, the role of caspase-4 in LPS-induced senescence is independent of its catalytic activity.

### The caspase-4 noncanonical inflammasome is induced and assembled during oncogene-induced senescence

**Fig. 4:**
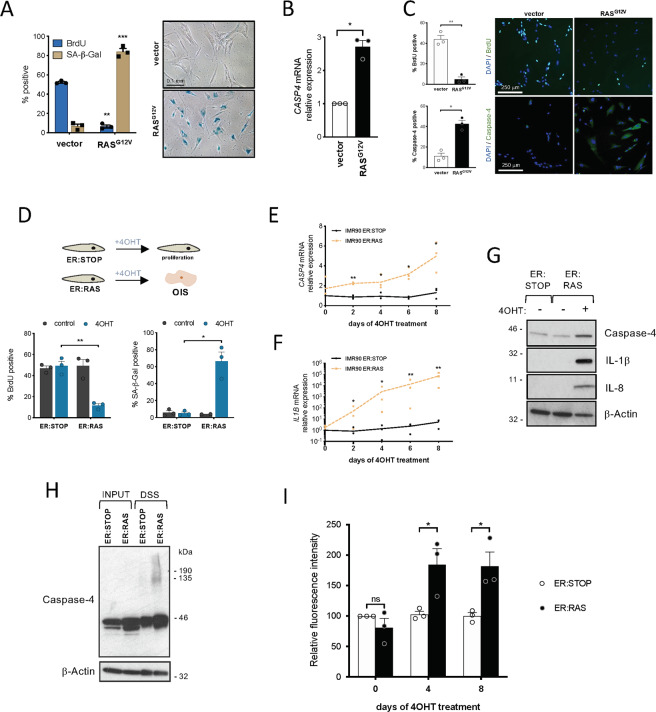
The caspase-4 noncanonical inflammasome is activated in oncogene-induced senescence. **A** IMR90 cells were infected with *RAS*^G12V^ expression vector to induce OIS. BrdU incorporation and SA-β-Galactosidase activity were measured 4 days after equal number of cells were seeded (left). Representative images (right) for SA-β-Galactosidase activity are shown. Graph bars, error bars and dots represent respectively the mean ± s.e.m. and the individual values of 3 independent experiments. Statistical analysis was performed using two-tailed Student’s *t* test. **B** *RAS*^G12V^-OIS was induced as in (**A**) and *CASP4* mRNA relative expression was quantified by RT-qPCR. Graph bars, error bars and dots represent respectively the mean ± s.e.m. and the individual values of 3 independent experiments. Statistical analysis was performed using two-tailed Student’s *t* test. **C** *RAS*^G12V^-OIS was induced as in (**A**) and BrdU incorporation and caspase-4 protein levels were measured by immunofluorescence in *RAS*^G12V-^OIS and control cells 4 days after equal number of cells were seeded. Graph bars, error bars and dots represent respectively the mean ± s.e.m. and the individual values of 3 independent experiments. Statistical analysis was performed using two-tailed Student’s *t* test. **D** IMR90 cells were infected with a control (ER:STOP) or an ER:RAS vector. Upon addition of 4OHT, ER:RAS cells undergo OIS. OIS can be detected by a reduction in BrdU incorporation (lower left) measured by immunofluorescence 5 days after 4OHT addition and an increase in SA-β-Galactosidase activity (lower right) one week after 4OHT addition. Graph bars, error bars and dots represent respectively the mean ± s.e.m. and the individual values of 3 independent experiments. Statistical analysis was performed using two-tailed Student’s *t* test. **E** Time-course experiment of *CASP4* mRNA relative expression in OIS. IMR90 ER:STOP and ER:RAS cells were treated with 4OHT and *CASP4* mRNA relative expression was quantified by RT-qPCR 0, 2, 4, 6 and 8 days after 4OHT addition. Graph lines and dots represent respectively the mean and the individual values of 3 independent experiments. Statistical analysis was performed using two-tailed Student’s *t* test. **F** Time-course experiment of *IL1B* mRNA relative expression in OIS. IMR90 ER:STOP and ER:RAS cells were treated with 4OHT and *IL1B* mRNA relative expression was quantified by RT-qPCR 0, 2, 4, 6, and 8 days after 4OHT addition. Graph lines and dots represent respectively the mean and the individual values of 3 independent experiments. Statistical analysis was performed using two-tailed Student’s *t* test. **G** IMR90 ER:STOP and ER:RAS were treated or not with 4OHT during eight days. Caspase-4, IL-1β and IL-8 protein levels were analyzed by immunoblotting. **H** To detect caspase-4 oligomers, IMR90 ER:STOP and ER:RAS cells were treated with 4OHT for five days, then cells were collected and subjected to disuccinimidyl suberate (DSS) crosslinking. After SDS-PAGE separation, DSS-cross-linked samples were probed for caspase-4 by immunoblotting. (**I**) Caspase-4 activity in OIS was measured using a fluorometric assay. IMR90 ER:STOP and ER:RAS cells were treated with 4OHT and LEVD-AFC cleavage was measured in low serum (0.5% FBS) cultured cells 0, 4, and 8 days after 4OHT addition. Graph bars, error bars and dots represent respectively the mean ± s.e.m. and the individual values of 3 independent experiments. Statistical analysis was performed using two-tailed Student’s t test. ***P < 0.001, **P < 0.01, and *P < 0.05 and ns, not significant. Scale bar = 250 μm as indicated.

Next, the role of inflammatory caspases in oncogene-induced senescence (OIS) was investigated. To induce OIS, *HRAS*^*G12V*^ (hereafter *RAS*^*G12V*^) was constitutively overexpressed in IMR90 human fibroblasts. *RAS*^*G12V*^ overexpression reduced cell proliferation and increased SA-β-Galactosidase activity (Fig. 4A). Coinciding with the upregulation of the cell cycle inhibitors p21^CIP1^, p16^INK4a^, and p15^INK4b^, caspase-4 expression was increased both at the mRNA and protein levels upon *RAS*^*G12V*^ overexpression (Fig. S4A, B and Fig. 4B, C). Next, we took advantage of an inducible system extensively employed by us and others to exert tight control over the onset of senescence [25]. In this system, a mutant form of the estrogen receptor (ER) ligand-binding domain is fused to the protein of interest (RAS); consequently, ER:RAS cells undergo OIS after the addition of 4-hydroxytamoxifen (4OHT) (Fig. 4D). As expected, IMR90 ER:RAS cells underwent cell proliferation arrest and showed increased SA-β-Galactosidase activity compared to non-treated IMR90 ER:RAS upon 4OHT addition (Fig. 4D). A time-course experiment using this system revealed that *CASP4* mRNA levels increase in parallel to the exponential increase in *IL1B* mRNA expression in cells undergoing OIS (Fig. 4E, F), and in caspase-4 protein (Fig. 4G). Also, we observed caspase-4 induction in paracrine senescence, and senescence induced by DNA damage with etoposide (Fig. S4C, D). Oligomerization of caspase-4 protein is essential for its activity [18]. These caspase-4 oligomers can be revealed in lysates crosslinked with disuccinimidyl suberate (DSS) as high-weight migratory bands in a western blot [26]. Endogenous caspase-4 oligomerization was detected in IMR90 ER:RAS senescent cells but not in control cells from 3 days after 4OHT treatment (Fig. 4H and Fig. S4E). Moreover, caspase-4 proteolytic activity was also increased in IMR90 ER:RAS cells 4 and 8 days after the addition of 4OHT (Fig. 4I). Altogether these results demonstrate that caspase-4 expression is induced and the noncanonical inflammasome is assembled in OIS.

### The caspase-4 noncanonical inflammasome is required for inflammatory signaling in OIS

**Fig. 5:**
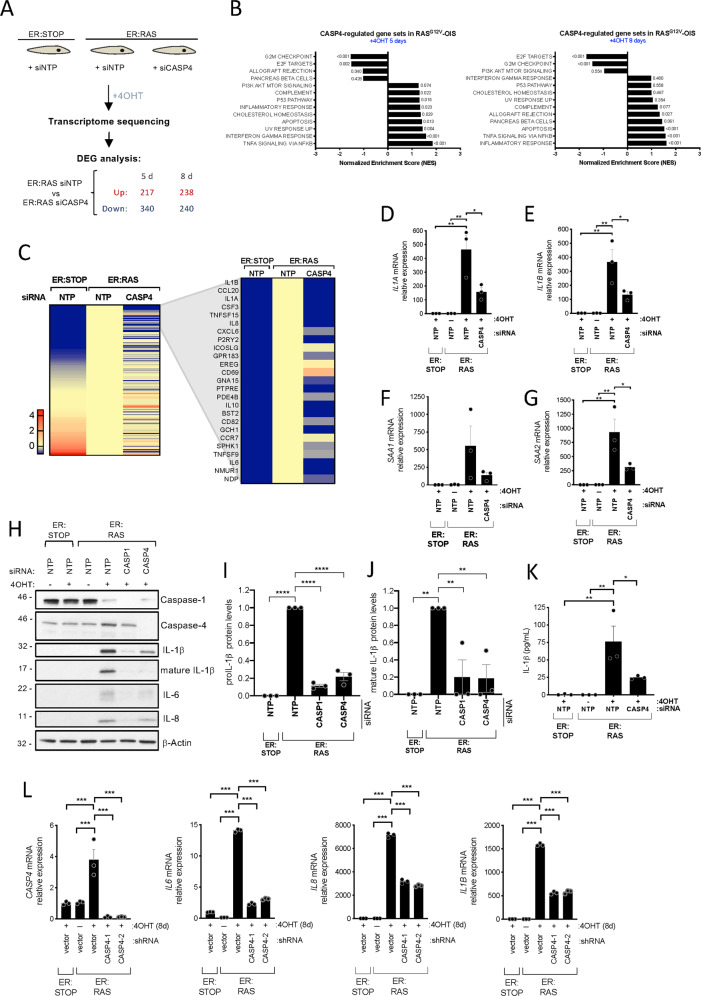
Caspase-4 activation controls the proinflammatory SASP. **A** Schematic diagram of the experimental approach. ER:STOP and ER:RAS IMR90 cells were targeted with either control (non-targeting pool, siNTP) or *CASP4*-targeting pool siRNA (siCASP4). All cells were treated with 4OHT from day 0. RNA was extracted 5 and 8 days after the addition of 4OHT and three independent biological replicates were subjected to transcriptomic analysis. Differentially expressed gene (DEG) analysis was performed and the number of significant upregulated and downregulated genes 5 and 8 days after the addition of 4OHT upon *CASP4*-targeting in ER:RAS cells is shown. **B** Normalized Enriched Scores (NES) of a set of 50 curated hallmark gene signatures were calculated based on the DEG analysis performed between control and *CASP4*-knockdown ER:RAS samples after 5 and 8 days of 4OHT treatment. Gene sets with a false discovery rate (FDR) q-value of ≤ 0.25 at least in one of the timepoints are shown. P-values for each gene set are indicated next to the corresponding bar. **C** Heatmap of the log2FC values of all 175 genes included in the “INFLAMMATORY RESPONSE” GSEA gene set of control ER:STOP and *CASP4*-knockdown ER:RAS compared to control ER:RAS after 5 days of 4OHT treatment. The top 25 differentially expressed signature genes in *RAS*^G12V^-OIS are zoomed in. **D** *IL1A* and (**E**) *IL1B* mRNA relative expression levels were quantified by RT-qPCR after 5 days of 4OHT treatment in ER:STOP and ER:RAS cells transfected with the indicated siRNAs. Graph bars, error bars and dots represent respectively the mean ± s.e.m. and the individual values of 3 independent experiments. Statistical analysis was performed using one-way analysis of variance (ANOVA). **F** *SAA1* and (**G**) *SAA2* mRNA relative expression levels were quantified by RT-qPCR after 8 days of 4OHT treatment in ER:STOP and ER:RAS cells transfected with the indicated siRNAs. Graph bars, error bars and dots represent respectively the mean ± s.e.m. and the individual values of 3 independent experiments. Statistical analysis was performed using one-way analysis of variance (ANOVA). **H** IMR90 ER:STOP and ER:RAS cells were transfected with control (NTP), *CASP1* or *CASP4-*targeting siRNA and treated with 4OHT or not during 8 days as indicated. Lysates were subjected to immunoblotting analyses with the indicated antibodies. **I** and **J** IMR90 ER:STOP and ER:RAS cells transfected with control (NTP), CASP1 or CASP4-targeting siRNA and treated with 4OHT or during 8 days. Full-length IL-1β (**I**) and mature IL-1β (**J**) levels were analyzed by immunoblotting and band intensities were quantitated. Graph bars, error bars and dots represent respectively the mean ± s.e.m. and the individual values of 3 independent experiments. Statistical analysis was performed using two-tailed Student’s *t* test. **K** Secreted IL-1β was quantified by ELISA in IMR90 ER:STOP and ER:RAS cells were treated or not 8 days with 4OHT as indicated. Graph bars, error bars and dots represent respectively the mean ± s.e.m. and the individual values of 3 independent experiments. Statistical analysis was performed using one-way analysis of variance (ANOVA). **L** IMR90 ER:STOP and ER:RAS cells were transduced with an empty retroviral vector (vector) or two different shRNAs targeting *CASP4* (shCASP4) and treated with 4OHT or not as indicated during 8 days. *CASP4, IL6, IL8, and IL1B* mRNA relative expression levels were quantified by RT-qPCR. ****P < 0.0001, ***P < 0.001, **P < 0.01, and *P < 0.05.

Next, a functional role for caspase-4 in OIS was investigated in our IMR90 ER:RAS system. We decided to use siRNA-pools of 4 siRNAs targeting *CASP4* for our global expression analysis to dilute potential off-target effects of the individual sequences occurring at higher siRNA concentrations. Global changes in mRNA expression upon *CASP4* depletion were analyzed. IMR90 ER:STOP and ER:RAS cells were transfected with control and *CASP4*-targeting small-interfering RNA (siRNA), samples were collected 5 and 8 days after 4OHT addition as it is immediately after caspase-4 activation and when a full SASP is developed respectively, and subjected to transcriptomic analysis (Fig. 5A)(Dataset 1). Knockdown control of the experiment was performed by mRNA expression analysis for *CASP4*, showing a substantial reduction of its expression upon siRNA targeting at both time points (Fig. S5A). Furthermore, *CASP4* was the top downregulated gene in *CASP4* siRNA-targeted compared to non-target siRNA control RAS^G12V^ cells both 5 and 8 days after the addition of 4OHT (Fig. S5B). Similarities between replicates and differences between conditions were confirmed by principal component analysis visualization and heatmap sample clustering (Fig. S5C, D). Differentially expressed gene analysis identified 557 and 478 genes significantly differentially expressed (FDR 10%) upon *CASP4* knockdown, of which 340 and 240 were induced in a *CASP4* dependent fashion in *RAS*^*G12V*^-OIS cells 5 and 8 days after 4OHT addition respectively (Fig. 5A). Gene set enrichment analysis (GSEA) of 50 hallmark gene sets of the transcriptomic data showed a CASP4 dependent regulation in *RAS*^*G12V*^-OIS cells of gene sets related to inflammatory processes, including TNF-α signaling and interferon responses, both 5 and 8 days after the addition of 4OHT (Fig. 5B). Enrichment plots of the gene signature hallmark “INFLAMMATORY RESPONSE” showed a positive correlation of this gene set expression with *CASP4* (Fig. S5E). Plotting a heatmap of the fold change values of control IMR90 ER:STOP and *CASP4* knockdown vs control IMR90 ER:RAS cells of all genes included in the “INFLAMMATORY RESPONSE” gene set revealed a pattern by which the increased expression of inflammatory-related genes in senescence is abrogated if *CASP4* is targeted, including SASP factors (Fig. 5C). Changes in the expression of *IL1A* and *IL1B* were validated by RT-qPCR (Fig. 5D, E). The serum amyloid A (SAA) proteins SAA1 and SAA2 belong to a family of apolipoproteins known to activate innate and adaptive immune cells and have recently been identified as SASP factors [14]. Of note, the expression of *SAA1* and *SAA2* was also decreased when *CASP4* was targeted in OIS (Fig. 5F, G). Targeting *CASP4* also reduced the amount of intracellular IL-1α, IL-1β, IL-6, and IL-8 protein to a similar extent than *CASP1* targeting (fig. S5F). Moreover, the levels of intracellular mature IL-1β were also significantly and similarly reduced when either *CASP1* or *CASP4* were targeted (Fig. 5H, I, J). Furthermore, the concentration of secreted IL-1β was significantly reduced in conditioned media of RAS^*G12V*^ induced cell cultures when *CASP4* was targeted (Fig. 5K). To confirm the specificity of the reagent used in our global gene expression analysis, we compared the siRNA pool consistent in 4 different siRNA used in our study with two of the individual siRNAs targeting *CASP4*, showing that all reagents reduced *CASP4* and *IL1B* mRNA and protein expression in IMR90 ER:RAS cells when compared to controls, confirming the specificity of the reagent used (Fig. S5G). Additionally, *IL1B*, *IL6,* and *IL8* mRNA induction in OIS were also impaired by knocking down *CASP4* expression with two retroviral shRNA vectors, increasing the specificity and robustness of the data (Fig. 5L). Overall, these results suggest that caspase-4 contributes to caspase-1 mediated SASP activation in OIS.

The SASP can induce senescence in adjacent growing cells through paracrine signaling, which is dependent on IL-1 signaling [5]. Because *CASP1* and *CASP4*-targeting reduced the production of several SASP factors and, in particular, limited the secretion of IL-1β, we next examined whether inflammatory caspases are implicated in SASP induction during paracrine senescence (Fig. S5H). Conditioned media from IMR90 ER:RAS senescent cells added to growing IMR90 fibroblasts produced the induction of *IL1A, IL1B*, *IL8,* and *IL6*, which was impaired when *CASP4* was targeted (Fig. S5H). In contrast, *CASP1* targeting did not affect the induction of the paracrine SASP (fig. S5H). Overall, these data suggest that the caspase-4 noncanonical inflammasome controls SASP activation during paracrine senescence. Altogether, these results indicate that the caspase-4 noncanonical inflammasome is a critical regulator of the SASP in OIS.

### Gasdermin-D is dispensable for SASP control in OIS

We then investigated the role of gasdermin-D in SASP during OIS. While mRNA levels remained unaffected (Fig. S6A), gasdermin-D was found to be cleaved during OIS (Fig. S6B, C), indicating some inflammasome activity over gasdermin-D in the process. However, cell death is not a hallmark of cellular senescence. Moreover, senescent cells are resistant to cell death, which can be induced using so-called senolytic drugs, such as the cardiac glycoside ouabain [27]. In our experimental settings, we did not observe increased cell death compared to controls upon Ras^G12V^ activation, but cell death was induced in senescent cells upon ouabain treatment (Fig S6D), suggesting that pyroptosis is not operating during OIS despite increasing gasdermin-D cleavage. Moreover, in contrast to *CASP1* or *CASP4*-targeting, *GSDMD* knockdown did not impair *IL1A*, *IL1B*, *IL8*, or *IL6* mRNA induction (Fig. S6E, F). Whereas targeting either *CASP1* or *CASP4* resulted in a significantly lower concentration of IL-1β in conditioned media from OIS cells, *GSDMD* knockdown did not alter IL-1β secretion (Fig. S6G), suggesting that IL-1β secretion is dependent on caspase-1 and caspase-4 but independent on gasdermin-D in OIS. Furthermore, overexpression of the catalytically inactive mutant *CASP4*^*C258A*^ during OIS did not oppose *IL1A* and *IL1B* expression, suggesting that CASP4 catalytic activity is dispensable for the SASP (Fig S6H, I). Thus, our results suggest that gasdermin-D cleavage and pyroptosis are not contributing significantly to OIS, and that an inhibitory mechanism may operate to prevent pyroptotic cell death during OIS.

### The caspase-4 noncanonical inflammasome contributes to the arrest in cell proliferation in OIS

**Fig. 6:**
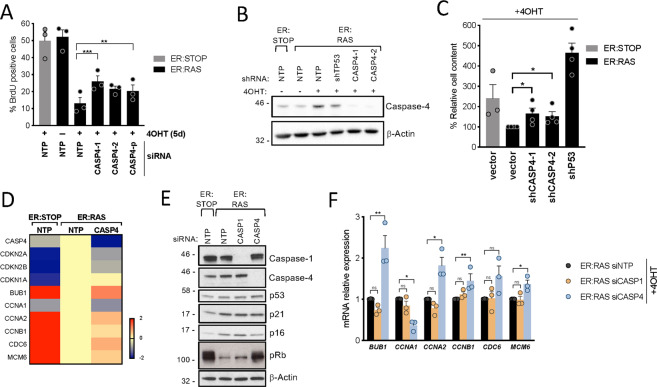
Caspase-4 contributes to the arrest in cell proliferation in OIS. **A** IMR90 ER:STOP and ER:RAS cells were transfected with control (NTP), two individual *CASP4*-targeting siRNAs (CASP4-1 and CASP4-2) or a pool of 4 different siRNA sequences targeting *CASP4* (CASP4-p), and treated with 4OHT or not as indicated. BrdU incorporation was measured by immunofluorescence 5 days after 4OHT addition. Graph bars, error bars and dots represent respectively the mean ± s.e.m. and the individual values of 3 independent experiments. Statistical analysis was performed using two-tailed Student’s *t* test. **B**, **C** IMR90 cells were transduced with an empty retroviral vector (vector) or shRNAs targeting either *CASP4* (shCASP4) or *TP53* (shP53). **B** Lysates were subjected to immunoblotting analyses with the indicated antibodies eight days after 4OHT addition. **C** On day 0, equal number of cells were subjected to 4OHT treatment. Fifteen days after 4OHT addition, plates were fixed and stained with crystal violet. Crystal violet was extracted and used to quantify cell content. Graph bars, error bars and dots represent respectively the mean ± s.e.m. and the individual values of 4 independent experiments. Statistical analysis was performed using two-tailed Student’s *t* test. **D** Related to Fig. 5A. DEG analysis was performed between control ER:STOP and *CASP4*-knockdown ER:RAS compared to control ER:RAS after 5 days of 4OHT treatment. Heatmap of the log2FC values from the indicated genes. **E** IMR90 ER:STOP and ER:RAS cells were transfected with control (NTP), *CASP1* or *CASP4*- targeting siRNAs and treated with 4OHT during 4 days. Cell lysates were subjected to immunoblotting analyses with the indicated antibodies. **F** IMR90 ER:RAS cells were transfected with control (NTP), *CASP1* or *CASP4*-targeting siRNA and mRNA relative expression of the indicated genes was quantified by RT-qPCR after 5 days of 4OHT treatment. Graph bars, error bars and dots represent respectively the mean ± s.e.m. and the individual values of 3 independent experiments. Statistical analysis was performed using one-way analysis of variance (ANOVA). ***P < 0.001, **P < 0.01, *P < 0.05 and ns, not significant.

To investigate the role of the noncanonical inflammasome in regulating cell proliferation in OIS, we targeted *CASP4* using RNAi. *CASP4*-targeting with siRNA transfection significantly rescued early cell proliferation arrest during OIS (Fig. 6A) and increased the total cell content in a long-term cell growth assay using shRNA retroviral vectors (Fig. 6B, C). Moreover, targeting *CASP4* during OIS modestly but significantly decreased SA-β-Galactosidase activity (Fig. S6J). A GSEA of 50 hallmark gene sets showed a negative regulation of *CASP4* in *RAS*^G12V^-OIS of the gene signatures “G2M CHECKPOINT” and “E2F TARGETS”, and the expression of the *CDKN2A* (p16^INK4a^-p14^ARF^) and *CDKN2B* (p15^INK4b^) locus, but not p21^CIP1^ (Figs. 5B, 6D, and Fig. S6K). Indeed, targeting *CASP4* reduced p16^INK4a^ expression and rescued the phosphorylation of pRb (Fig. 6E and Fig. S6L) and resulted in a transcriptional increase in the levels of E2F target genes (Fig. 6F), suggesting a role for caspase-4 regulating cell proliferation by controlling p16^INK4a^ and p15^INK4b^ expression in OIS. Of note, activation of caspase-4 by intracellular LPS resulted in increased levels of E2F target genes (Fig. S6M). Finally, similarly to SASP regulation, *GSDMD* targeting did not alter the arrest in cell proliferation in OIS (Fig. S6N, O), suggesting that gasdermin-D has no significant role in OIS.

Altogether, these results suggest that caspase-4 contributes to the arrest in cell proliferation during senescence, impacting ultimately on the phosphorylation state of pRb resulting in transcriptional repression of E2F target genes.

### Caspase-11 is induced during cellular senescence in vivo

**Fig. 7:**
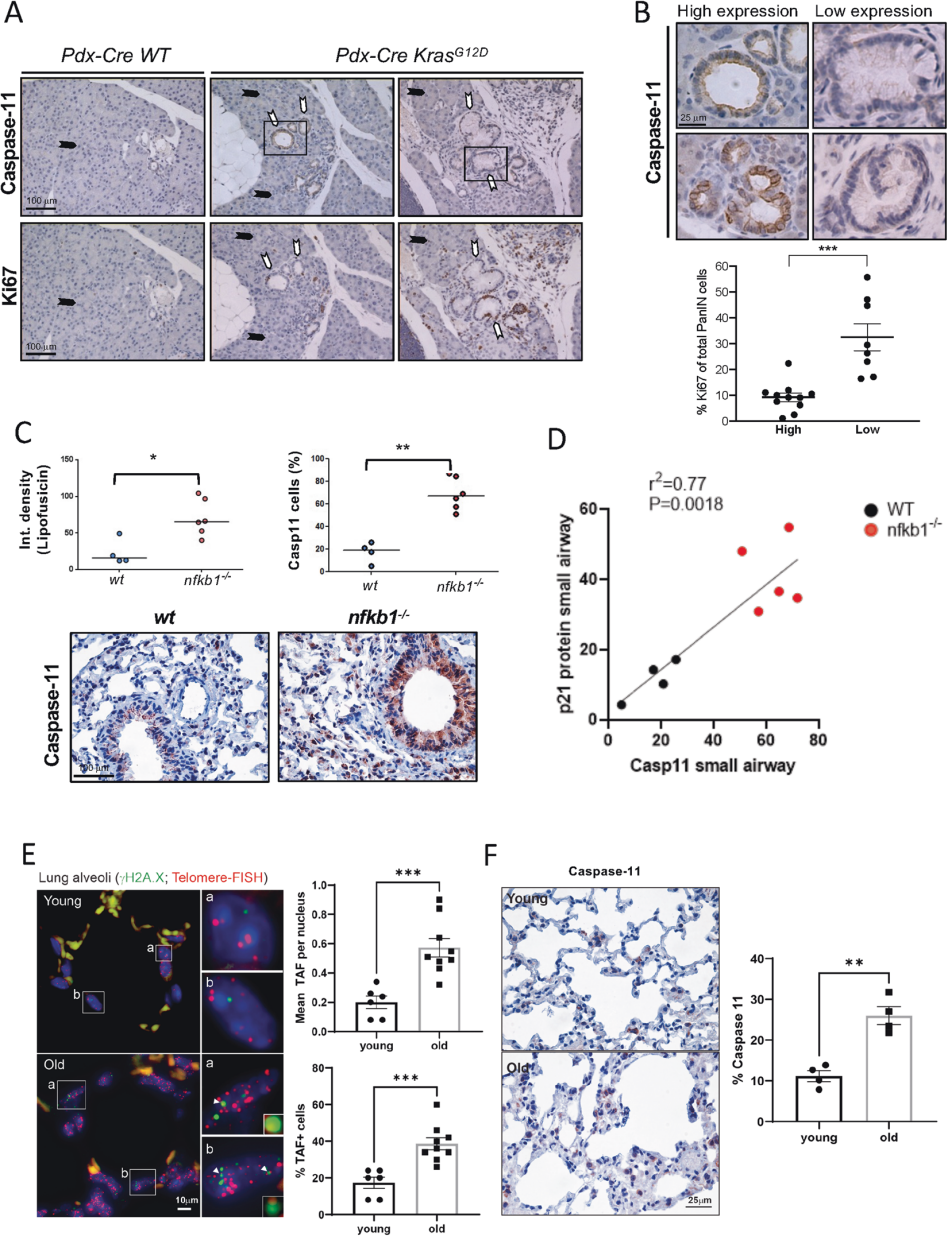
Caspase-11 expression is induced in senescence in vivo. **A** Immunohistochemistry showing Ki-67 and caspase-11 staining in sections from *Pdx-cre WT* and *Pdx-cre Kras*^*G12D*^ pancreas (left panels). Black arrows indicate acinar pancreatic cells, white arrows indicate PanIN cells. **B** Close up images showing PanINs with high and low expression of caspase-11. Quantification of Ki-67 positive cells of total PanIN cells from a total of 11 mice of 7 to 15 weeks of age. PanINs were classified according to the expression of caspase-11 as indicated. The percentage of Ki-67 positive cells was calculated scoring all cells of PanINs classified as high or low caspase-11 expression per mouse as indicated. Scatter plots were generated from total cells from high and low caspase-11 expressing PanINs with individual points representing the mean Ki-67 percentage positivity for each mouse, with horizontal lines representing group mean and s.e.m. Statistics: Mann–Whitney *U* test. ***p < 0.001. **C** Analysis of caspase-11 expression was conducted by immunohistochemistry in lung sections from wild type (WT) or *nfkb1* knock out mice (*nfkb1*^*-/-*^) at 9.5 months of age. 10–15 random images were captured per mouse and average percentage positivity calculated for airway epithelial compartments. Scatter plots represent mean percentage positivity for each animal with horizontal line representing group median. Broad-band autofluorescence (an indicator of lipofuscin accumulation) was acquired from paraffin-embedded sections excited at 458 nm with fluorescence emission captured above 475 nm using a fluorescence microscope (Leica DM550B). Fluorescence intensity was analyzed using ImageJ. At least 10 small airways were analyzed per mouse and an average intensity calculated per animal. Scatter plots represent average value per animal with the horizontal line representing group median. Statistics: Mann–Whitney *U* test. *p < 0.05, **p < 0.01. Representative images of caspase-11 staining by immunohistochemistry in airway epithelial cells from wt and *nfkb1-/-* mice, captured using x40 objective. **D** Linear regression graph between representing caspase-11 expression from samples in (**C**), and p21 expression from the same samples, which were previously published in Hari et al. [14]. **E** Representative image of Immuno-FISH for γH2A.X (green) and telomeres (red) in lung alveoli cells from 6.5-month and 24-month-old mice captured using X100 oil objective. Arrows point to γH2A.X foci co-localizing with telomeres (TAF). Statistics: Mann–Whitney *U* test. ***p < 0.001. **F** Analysis of caspase-11 expression by immunohistochemistry in lung sections of wt mice at 6.5 months of age (Young) and 24 months of age week (Old). Scatter plots were generated from 10–15 random images captured per animal with individual points representing mean percentage positivity for each mouse with horizontal line representing group median. Statistics: Mann–Whitney U test. **p < 0.01. Representative images of caspase-11 staining by immunohistochemistry (positive, brown; negative, blue) in alveolar cells from wt mice 6.5 and 24 months of age, captured using x40 objective. Scale bar = 100 μm and 25 μm as indicated.

We have shown that caspase-4 expression levels are critical in cellular senescence. To investigate noncanonical inflammasome expression in senescence in vivo, we used three well-characterized mouse models of senescence. We first analysed the caspase-4 murine homologous caspase-11 expression in a model of OIS in which conditional expression of *Kras*^*G12D*^ by *Pdx-CRE* induces Pancreatic Intraepithelial Neoplasia (PanIN) in the pancreas of mice [28] (Fig. 7A). To validate the antibody used in the analysis, we performed caspase-11 immunohistochemistry in MEFs transfected with siRNA against *CASP11* that were processed like tissue samples, showing a decreased caspase-11 signal when compared to cells transfected with nontargeting controls, indicating an optimal antibody specificity (Fig. S7A). We observed that low-grade PanINs stained positive for caspase-11 when compared to surrounding pancreatic acinar cells, higher grade PanINs, and in the ducts and acinar cells in wild-type mice (Fig. 7A). Importantly, quantification of Ki-67 staining in PanINs showed that the expression of caspase-11 was restricted to early senescent lesions with low proliferative index (Fig. 7B), indicating that caspase-11 expression correlates with lower cell proliferation in low-grade PanIN lesions.

We then investigated the expression of caspase-11 in two additional models of cellular senescence associated to aging in vivo. First, we used a mouse model of accelerated aging by constitutive activation of NF-κB by knockout of its regulator *nfkb1*^*-/-*^ (*p50*^*-/-*^), which shows senescence in different tissues including the lung [29]. In this model, lipofuscin accumulation has been shown to be associated with senescence and it is increased in lungs from *nfkb1*^*-/-*^ compared to wild-type mice (Fig. 7C). A similar increase in lipofuscin accumulation was detected in caspase-11 expressing cells (Fig. 7C). Furthermore, we found a correlation between p21 and caspase-11 in small airway epithelial cells from wild-type and *nfkb1*^*-/-*^ mice [14] (Fig. 7D), indicating that caspase-11 correlates with senescence markers in a model of accelerated aging in the mouse.

Then, we investigated caspase-11 expression in alveolar cell senescence during mouse natural organismal aging. Alveolar cells of aged mice (24 months) showed an increased number of Telomere-Associated DNA Damage Response (DDR) foci (TAFs) [29] (Fig. 7E), a marker of the accumulation of senescent cells in tissues, which we found that occurred concomitantly with the increase in caspase-11 (Fig. 7F). In summary, these results support the model that noncanonical inflammasomes contribute to senescence in vivo.

## Discussion

Here, we show that microbial pathogenic stress caused by cytoplasmic LPS accumulation and recognition by the caspase-4 noncanonical inflammasome induces a senescence response, in which sublethal levels of LPS activate the p16^INK4a^-pRb and p53- p21^CIP1^ tumor suppressor pathways (Fig. S7B). Interestingly, while LPS-mediated pyroptosis requires caspase-4 and the effector protein gasdermin-D but not p53, the senescence response requires the participation of p53. These results suggest a mechanism in which p53 controls the cellular stress responses to microbial infection downstream of caspase-4 until a certain threshold level in which gasdermin-D-dependent pyroptosis eliminates highly damaged cells, introducing a new context-dependent role for p53 in innate immune sensing [30]. Further research will be necessary to determine the functional role of senescence in response to infection.

Our results describe a new mechanism of senescence induction by cytoplasmic microbial sensing, adding to the essential triggers such as the DNA Damage Response, telomere attrition, oncogenic activation, mitochondrial damage, or ribosome biogenesis inactivation [1, 31]. We also show that both the expression and the assembly of the caspase-4 noncanonical inflammasome are triggered upon oncogene activation. Furthermore, we observe that caspase-4 is critical for SASP induction and contributes to the arrest in proliferation in OIS (fig. S7B). Thus, these results suggest that the role of caspase-4 in senescence is conserved in a sterile context and that there is crosstalk between antimicrobial immune responses and tumor suppression. Notably, the assembly of the caspase-4 inflammasome is an early event in OIS, peaking after day three to four after Ras activation (Fig. 4). Recent results suggest that OIS is a highly dynamic process, with distinct signaling waves contributing to the establishment of the senescent phenotype. Interestingly, the specific time point where caspase-4 is assembled coincides with the moment of transition to a proinflammatory, NF-κB -dependent SASP in OIS [32, 33]. It is plausible that the activation of the noncanonical inflammasome could play a critical role in this transition.

Unexpectedly, our results indicate that LPS-mediated caspase-4 induced senescence is not accompanied by robust activation of IL-1β and the SASP. Thus, our data indicates that caspase-4 functions in the arrest in cell proliferation is independent of its downstream effect on IL-1β activation, suggesting a split in tumor suppressive and proinflammatory functions. Interestingly, a significant SASP induction is only achieved when LPS stimulation and priming of the inflammasome with TLR2 ligands happen simultaneously, suggesting that caspase-4 controls the cell proliferation and the SASP by uncoupled mechanisms (Fig. S7). Besides, our data show that caspase-4 heavily influences the SASP induction during OIS, where sustained production of A-SAA signaling through TLR2 is critical to the SASP [14]. Thus, caspase-4 appears to be central regulating the induction of cellular senescence, the SASP, or pyroptosis in response to microbe-derived molecules and to sterile cellular stresses. Further research will be required to identify the nature of the signal responsible for caspase-4 activation during OIS.

Mechanistically, our data suggest that the role of caspase-4 in senescence is independent from its catalytic activity. Caspase-4 is a pattern recognition receptor that binds LPS directly;[18] therefore, it is plausible that upon ligand recognition, caspase-4 functions as a molecular platform for the activation of downstream senescence pathways in a proteolytic independent fashion. Our data suggest that pyroptosis is triggered only at a certain threshold of caspase-4 induction, suggesting a dose-dependent functional split between the caspase-4 pro-senescent and pyroptotic functions.

Our data reveal that the pyroptotic effector protein gasdermin-D also contributes to LPS-induced senescence. The cleavage of gasdermin-D triggers pyroptosis by inflammasomes; however, the proteolytically inactive mutant of caspase-4, which inhibits LPS-induced pyroptosis, can induce cellular senescence, which suggests that caspase-4 and gasdermin-D regulate LPS-induced senescence with a mechanism other than the cleavage and liberation of the gasdermin-D amino-terminal domain. In addition, the tumor suppressor protein p53 contributes to LPS-induced senescence, but it is dispensable for pyroptotic regulation, reinforcing the notion of diverging pathways controlling senescence and pyroptosis in response to cytoplasmic LPS.

In contrast to LPS-induced senescence, our results indicate that gasdermin-D is dispensable for OIS, indicating an alternative mechanism contributing to cellular senescence. Alternative mechanisms have been described upstream and downstream caspase-4 in response to distinct stressors. For example, it has been reported that caspase-4 can assemble an Apaf-1 pyoroptosome in response to mitochondrial permeability transition, activating an alternative effector pathway that results in caspase-3 and gasdermin-E dependent pyroptosis [34]. Hence, it is plausible that a similar mechanism distinct to gasdermin-D cleavage by caspase-4 operates in OIS. Furthermore, our data indicate that senescent cells resist cell death induced by gasdermin-D cleavage during OIS. Interestingly, continuous toll-like receptor stimulation has been shown to confer tolerance to inflammasome activation and resistance to pyroptosis in macrophages [35]. Importantly, we have seen previously that sustained TLR2 activation is critical to OIS;[14] thus, it is possible that sustained TLR2 signaling in OIS might similarly enhance tolerance to late inflammasome activation eliciting cell death resistance. Altogether, our data indicate a divergence between the mechanisms controlling pyroptosis and cellular senescence by noncanonical inflammasomes. Nevertheless, further investigation is needed to elucidate the specific mechanism of senescence induction by caspase-4 in LPS-induced senescence and OIS, the divergent roles of gasdermin-D in both processes, and the mechanism behind the potential pyroptosis resistance upon gasdermin-D cleavage in cellular senescence.

Inflammasomes have shown diverse pro- and antitumorigenic functions in cancer [36]. Our data suggest that caspase-4 activation is part of a homeostatic response to oncogenic stress. In agreement with this observation, we show that caspase-11 (caspase-4 homologous in mice) expression is induced following tumor initiation in a genetically engineered mouse model of pancreatic cancer, suggesting its activation in cancer initiation. Also, we observed induction during mouse aging. In recent years several strategies have been implemented to eliminate senescent cells or to modulate the activation of the SASP in antiaging and cancer therapies[37–39]. Furthermore, the pharmacological targeting and removal of senescent cells has been shown to improve homoeostasis following tissue damage and aging [40, 41]. Here we propose that manipulation of noncanonical inflammasomes could provide a new rationale for senotherapies targeting the SASP and inducing pyroptosis in cancer and aging.

## Materials and methods

### Cell culture

HEK293T, MEFs, CAPAN-1, PSN-1, A549, HCT116, THP-1, and IMR90 female human fetal lung fibroblast cells were obtained from American Type Culture Collection. All cell lines except THP-1 that were culture in RPMI-1640 Medium (Sigma), were maintained in Dulbecco’s Modified Eagle’s Medium (DMEM) (Sigma), supplemented with 10% Fetal Bovine Serum (FBS) (ThermoFisher) and 1% antibiotic-antimycotic solution (ThermoFisher). All cell lines were cultured at 37 °C with 5% CO_2_ and tested for mycoplasma on a regular basis. All cell lines were regularly tested for mycoplasma contamination using the Mycoalert Mycoplasma Detection Kit (Lonza). Cell counting and viability were performed Muse® Count & Viability Assay Kit in a Muse Cell Analyser (Merck Millipore).

### Chemical compounds and treatments

OIS was induced by treating IMR90 ER:RAS cells with 100 nM 4OHT (H7904, Sigma). IMR90 ER:RAS and control ER:STOP were maintained in standard media supplemented with 200 μg/ml geneticin (10131-027, ThermoFisher). To induce oncogene-induced senescence, IMR90 ER:RAS cells were treated with 100 nM 4-hydroxytamoxifen (4OHT) for 48 h, followed by 5 days in normal culture media, For DNA damage-induced senescence, IMR90 cells were treated with 100 μM Etoposide for 48 h. For noncanonical inflammasome activation and inflammasome priming experiments, ultrapure lipopolysaccharide (LPS) from E. coli 111:B4 (tlrl-3pelps, Invivogen), muramyldipeptide (MDP) (tlrlmdp, Tocris), Pam2CSK4 (4637, Tocris), Pam3CSK4 (4633, Tocris), Recombinant Human Apo-SAA (A-SAA)(300-13, Peprotech) and BSA (Sigma) were used. For priming time-course experiments, the following concentrations were used: LPS (1 μg/ml), MDP (1  μg/ml), Pam2CSK4 (50 ng/ml), Pam3CSK4 (500 ng/ml). To prime inflammasomes prior to LPS transfection, cells were treated with Pam2CSK4 (1 μg/ml) A-SAA (10 μg/ml) or BSA (10 μg/ml) for 3 h. Ouabain (Apexbio) (100 nM) was used as a senolytic.

### Cell quantification and viability

To determine the viable cell concentration of cultures, cells were washed and incubated with trypsin (ThermoFisher) for 5 min at 37 °C. Fully detached cells were collected by centrifugation, resuspended in Muse Count & Viability Reagent (Merck Millipore), and counted using the Muse Cell Analyzer (Merck Millipore). To determine cell viability, culture supernatants and attached cells were pooled together before centrifugation. Pellets were resuspended in Muse Count & Viability Reagent (Merck Millipore) and analysed using the Muse Cell Analyzer (Merck Millipore).

### LPS transfection

To electroporate LPS or MDP, the Neon Transfection System (MPK5000, Invitrogen) and the Neon Transfection System 100 μL Kit (MPK10025, Invitrogen) were used. Per each tip, 5 ×10^5 cells were transfected with the indicated amount of ultrapure lipopolysaccharide (LPS) from E. coli 111:B4 (tlrl-3pelps, Invivogen) or MDP (tlrlmdp, Tocris). Electroporation parameters were set at 1500 V, 30 ms and pulse number 1 for IMR90 cells and 1100 V, 20 ms and pulse number 2 for HEK293T cells. Once electroporated, the tip content was unloaded into a clean Eppendorf tube and tubes were centrifuged on a bench-top centrifuge at 3000 rpm 3 min. The supernatant was removed to avoid any traces of MDP or LPS in the extracellular media prior to plating. For CAPAN-1, PSN-1, A549, and HCT116, 1 to 10 μg/mL MDP and LPS were transfected in DMEM media with 10% FugeneHD (Promega).

### Conditioned medium for paracrine senescence transmission

For the production of conditional medium (CM), IMR90 ER:STOP and ER:RAS cells were cultured as previously described [25]. IMR90 ER:STOP and ER:RAS cells were cultured with DMEM supplemented with 100 nM 4OHT and 10% FBS for 4 days, and with DMEM 100 nM 4OHT and 1% FBS for 4 additional days. CM was filtered using 0.2 μm syringe filters (Millipore) and reconstituted with a solution of DMEM supplemented with 40% FBS at a 3:1 ratio.

### Generation of plasmids

Using standard retro-transcription procedures, total RNA extracted from IMR90 cells was converted into cDNA generating a human coding sequence (CDS) library. CASP1 and CASP4 CDS were amplified from the obtained library and cloned into the pMSCV-puro vectors. The caspase-4 catalytically dead C258A pMSCV-puro vector was generated from the wild-type pMSCV-puro-CASP4 through site-directed mutagenesis by PCR using the Q5 Site-Directed Mutagenesis Kit (E0554S, New England Biolabs). The CASP4 and GSDMD-targeting lentiviral vectors (pGIPZ) were purchased from Dharmacon. The CASP4-targeting retroviral vectors (pRS-shCASP4-1 and pRS-shCASP4-2) were generated inserting the oligonucleotides (+) GATCCCCCAACGTATGGCAGGACAAATTCAAGAGATTTGTCCTGCCATACGTTGTTTTTG and GATCCCCTAACATAGACCAAATATCCTTCAAGAGAGGATATTTGGTCTATGTTATTTTTG, respectively, into the pRS empty backgone following the pSuper RNAi System manual (OligoEngine) instructions. pLN-ER:RAS, LSXN-ER:Stop, MSCV-Ras^G12V^, pCMV-VSVG, and pUMVC3-gag-pol vectors have been described elsewhere [5].

### Retroviral and lentiviral production and infection

For retroviral production, 20 μg retroviral plasmid were cotransfected with 2.5 μg pCMV-VSVG envelope plasmid and 7.5 μg pUMVC3-gag-pol helper vector using polyethylenimine linear (Alfa Aesar) into HEK293T cells. For lentiviral production, 10 μg lentiviral plasmid were cotransfected with 2.5 μg pCMV-VSVG and 7.5 μg psPAX2 using polyethylenimine linear (Alfa Aesar) into HEK293T cells. Viral supernatant was collected from the HEK293T cells 2 days after transfection and passed through a 0.45 μm syringe filter (ThermoFisher). The viral supernantant was complemented with hexadimethrine bromide (Sigma) to a final concentration of 4 μg/mL. When performing retroviral infections, IMR90s were treated with fresh viral supernatant and subsequent viral supernatant collection and incubation of IMR90 cells was performed every 3 h until three rounds of infection were performed. For lentiviral infections, a single 3 h incubation with 1:10 dilution of viral supernatant was performed. In both cases viral supernatant was removed after the indicated rounds of infection, fresh media was added to IMR90 cells and, 2 days later, selection with puromycin (1 μg/ml) (A11138, ThermoFisher) was initiated. Before set-up, fresh standard media supplemented with the selection agent was added for over a week or until no alive cells were observed in control cells infected with a noncontaining selection marker vector.

### siRNA transfection

ON-TARGETplus siRNAs were obtained from Dharmacon. Sequences and IDs are detailed in Supplementary Table 2. For all transfections, 30 nM siRNA were incubated up to 1 h with Dharmafect 1 (Dharmacon, 1 μg/ml final use concentration) to allow the formation of siRNA:transfection agent complexes prior to transfection. On day 0, 200.000 IMR90 ER:STOP and ER:RAS cells were plated in each T-6 well, 4OHT was added and siRNA reverse transfections performed. Due to the transient nature of siRNA, cells were split 1:4 on day 3 and reverse transfections were repeated. To maintain the knockdown during 8 days, forward transfections were performed again on day 5.

### Total RNA preparation and quantitative reverse transcription-polymerase chain reaction (qRT-PCR)

Cell lysates were homogenized using QIAshredder (Qiagen) and RNA was extracted using the RNeasy Plus Mini kit (Qiagen). RNA was transformed into cDNA using qScript cDNA Supermix (Quanta Biosciences) following manufacturer’s instructions. To perform quantitative PCRs, samples were prepared in triplicates in 96-well plates. Each well contained 1 μL of cDNA, 200 nM forward primer, 200 nM reverse primer, 1x SYBR Select Master Mix (Applied Biosystems) and up to 20 μL of ultrapure DNase/RNase-free distilled water (ThermoFisher). Plates were loaded into a StepOnePlus Real-Time PCR System (ThermoFisher) and the following PCR cycling parameters were used: 10 min at 95 °C; 40 cycles of 15 s at 95 °C, 30 s at 60 °C and 15 s at 72 °C; 15 s at 95 °C. Data were analyzed using the double Delta Ct method. The housekeeping gene ACTB was used to normalize data. Primers are specified in the Supplementary Table 3.

### Immunofluorescence and high-content microscopy

Cells were fixed with 4% paraformaldehyde (FD NeuroTech) in PBS during 45 min. All incubations were performed at room temperature and on an orbital shaker. To permeabilize cells, cells were incubated with 0.2% Triton-X100 in PBS for 10 min. Cells were blocked with immunofluorescence blocking buffer (1% Bovine Serum Albumin (BSA) and 0.2% Fish Gelatin in PBS). Primary and secondary antibodies were diluted in immunofluorescence blocking. Anti-BrdU primary solution was supplemented with 0.5 U / μL DNAse (Sigma) and 1 mM MgCl2 to improve anti-BrdU access to DNA-bound BrdU. Nuclei were stained with 1 μg/mL 4ʹ,6-diamidino-2-phenylindole (DAPI) (Molecular Probes). Antibodies are listed in Supplementary Table 1. Immunofluorescence was analyzed using the High-Content Image Acquisition and Analysis software (Molecular Devices) as previously described [25]. One-wavelength images of the same frame were merged using the software Fiji (ImageJ).

### Western blot analysis

Whole cells were lysed in 1X Cell Lysis Buffer Cell (Cell Signaling) supplemented with cOmplete EDTA-free Protease Inhibitor Cocktail (Roche). Protein concentration was determined by the Bradford assay using the Bradford reagent (Biorad) and BSA pre-set standards (ThermoFisher) to construct a standard curve. To prepare samples for sodium dodecyl sulfate-polyacrylamide gel electrophoresis (SDS-PAGE), 15 μg of protein were mixed with 6 μL 6x reducing Laemmli SDS sample buffer (Alfa Aesar) in a final volume of 36 μL. Samples were boiled 5 min at 95 °C and loaded in precast Novex Tris-Glycine gels (Invitrogen). Precast gels were run in an XCell SureLock™ Mini-Cell Electrophoresis tank (ThermoFisher) at 100–140 V. Proteins were transferred into nitrocellulose membranes using the iBlot Gel Transfer Device (ThermoFisher). Membranes were blocked in Tris-buffered saline (TBS) buffer (25 mM Tris-HCl + 137 mM NaCl + 2.7 mM KCl, pH7.4) supplemented with 5% nonfat milk 1 h at room temperature on a rocking shaker. Primary and secondary antibodies (described Supplementary Table 1) were diluted in TBS 5% milk buffer. To visualize bands, membranes were incubated with enhanced chemiluminescence solution (GE Healthcare) and exposed to X-ray films (GE Healthcare). Band signal intensities were quantified using the Fiji ImageJ software.

### Caspase-4 fluorometric activity assay

To measure LEVD-AFC cleavage, the Caspase-4 Fluorometric Assay kit (ab65658, Abcam) was used following manufacturer’s instructions. 2 ×10^6 IMR90 ER:STOP or ER:RAS cells were lysed in 50 μL cell lysis buffer. The assay was conducted in black sterile 96-well polystyrene plate (ThermoFisher). Fluorescence was measured (excitation filter: 400 nm; emission filter: 505 nm) using an Infinite^®^ 200 PRO (Tecan) plate reader.

### Detection of caspase-4 oligomerization

Fresh IMR90 ER:STOP or ER:RAS cell pellets were resuspended in 0.5 ml of ice-cold buffer A (20 mM HEPES-KOH, pH 7.5; 10 mM KCl; 1.5 mM MgCl2; 1 mM EDTA; 1 mM EGTA; 320 mM sucrose), lysed by shearing 10 times through a 25-gauge needle, and centrifuged 8 min at 1.800 g at 4 °C. At this point, 30 μL of lysates were kept as input controls. Remaining supernatants were diluted with 1 volume of CHAPS buffer (20 mM HEPES-KOH, pH 7.5; 5 mM MgCl2; 0.5 mM EGTA; 0.1 mM PMSF; 0.1% CHAPS) and centrifuged 8 min at 5000 x *g*. Supernatants were discarded and pellets were resuspended in 50 μL of CHAPS buffer containing 4 mM of disuccinimidyl suberate (DSS) during 30 min at room temperature to cross-link proteins. Then, samples were centrifuged 8 min at 5,000 x *g* at 4 °C, supernatants discarded and pellets resuspended in 60 μL of protein loading buffer (25 mM Tris-HCl, pH 6.8; 1% SDS; 10% glycerol; 6.25 mM EDTA; 0.01% bromophenol blue). Samples were heated for 2 min at 90 °C and 18 μL of resuspended cross-linked pellets were loaded onto a 4–12% precast Novex Tris-Glycine gels (Invitrogen). Further immunodetection of caspase-4 was performed following standard western blotting procedures.

### Determination of IL-1β content in conditioned media

Conditioned media was collected, centrifuged 10 min at 1000 rpm at 4 °C and transferred to a clean tube. Released IL-1β was quantified using the Human IL-1 beta ELISA Ready-Set-Go! Kit (15581087, ThermoFisher) following the manufacturer’s instructions. Conditioned media IL-1β concentrations were deducted interpolating the data from the standard curve, as previously described [42].

### Cell proliferation assays

5-bromo-2′-deoxyuridine (BrdU) incorporation was used to measure the number of cells actively replicating DNA. Cells were incubated with 10 μM BrdU (85811, Sigma) for 16 to 18 h. Cells were stained for immunofluorescence and high-content microscopy as described.

To analyze long-term growth, low equal amounts of cells were plated in 10 cm diameter dishes. Media was changed every 3 days and cells were fixed two weeks after initial seeding with 0.5% glutaraldehyde (Sigma) in PBS for 20 min and left drying overnight. Dishes were stained with 0.2% crystal violet for 3 h, washed twice with tap water and dried. To quantify cellular mass, cell-bound crystal violet was extracted in 10% acetic acid, equal amounts were transferred to a spectrophotometer-compatible 96-well plate and absorbance was read at 595 nm.

### SA-β-Galactosidase assay

5 ×104 IMR90 cells per well were seeded in 6-well plates. Four days later, cells were fixed with 0.5% glutaraldehyde (Sigma) in PBS during 10 min. Fixed cells were washed three times with PBS 1 mM MgCl2 pH 5.7, before adding to each well 2 mL of prewarmed X-Gal staining solution (2 mM MgCl2, 5 mM K4Fe(CN)6 • 3H2O, 5 mM K3Fe(CN)6, 1 mg/mL X-Gal solution ready to use (R0941, ThermoFisher) in PBS). Plates were incubated for 2–24 h at 37 °C, washed and imaged. SA-β-Gal activity positive and negative cells were quantified using FIJI/ImageJ.

### AmpliSeq transcriptome profiling

RNA quality was assessed on the Bioanalyser 2100 Electrophoresis Instrument (Agilent) with the RNA 6000 Nano Kit (5067-1511, Agilent). Samples were quantified using the Qubit 2.0 fluorometer and the Qubit RNA Broad Range assay.10 ng of RNA was reverse-transcribed to cDNA, and target genes were amplified for 12 cycles of PCR using the Ion AmpliSeq Human Gene Expression Core Panel (A26325, Thermofisher). This panel contains a pool of 20,802 amplicons (41,604 primers) of approximately 150 bases in length. Ion Torrent sequencing adapters and barcodes Ion XpressTM Barcode Adapters (Ion XpressTM Barcode Adapters) were ligated to the amplicons and adapter-ligated libraries were purified using AMPure XP beads. Libraries were quantified by qPCR and diluted to 100 pM before being combined in equimolar pools of 8 per each Ion PI Chip Kit v3 (ThermoScientfic). Sequencing was performed using the Ion PI Hi-Q Sequencing 200 Kit (A26433, ThermoFisher). Sequence reads were mapped to the hg19_AmpliSeq_Transcriptome_ERCC_v1.fasta reference. BAM files were generated using the Torrent Suite software v 5.2.0 (ThermoFisher). Differentially expressed gene (DEG) analysis was performed with the DESeq2 package v.1.20.0 (Bioconductor). Gene Set Enrichment Analysis (GSEA) was performed using the Broad Institute GSEA software v3.0. DEG-obtained log2FC values were used as inputs for the GSEA. Molecular signatures were obtained from MSigDB v.6.2 (UC San Diego / Broad Institute). The transcriptomic data generated during this study is available at the Gene Expression Omnibus (GEO) database with number GSE188373.

### Experiments with mice

Experiments were performed according to UK Home Office regulations. Mice carrying a conditional *Pdx1–Cre Kras*^*G12D/+*^ allele were used and have been described previously [28]. Sections of formalin-fixed paraffin-embedded mouse pancreas from 6 to 14-week-old mice were stained with antibody against Ki67 and Caspase-11. Caspase-11 signal was used to classify high and low Caspase-11 expressing PanIN. Aging and inflammation experiments were carried out on male wild-type C57BL/6 mice or male *nfkb1*^*-/-*^ mice on a pure C57BL/6 background at 6.5, 9.5, and 24 months of age. Mice were randomly assigned to young or old groups in aging experiments. Sample sizes were based in standard protocols in the field. Experiments were blinded to the person performing marker analysis.

### Immunohistochemistry

For the Pdx1–Cre Kras^G12D/+^ mice (PanIN), Using EnVision^TM^ + Dual Link system-HRP (DAB + ) kit (K4065, Dako), sections of formalin-fixed paraffin-embedded mouse pancreas were stained with antibody against Ki67 (ab21700, Abcam). The total number of Ki67 positive cells per PanIN, and the total cells per PanIN were counted, and thus the percentage of Ki67 positive cells per PanIN was calculated. The mean score for each mouse was calculated and these scores were plotted scatter plot. Consecutive sections were stained with antibodies against Caspase-4/11 (bs-6858R, Bioss). The stainings were examined and classified for high or low expression of the respective antibodies, and each structure compared with the Ki67 percentage.

For nfkb1-/- and aging mice analysis, sections were dewaxed in histoclear (5 min), rehydrated through graded ethanol solutions (100, 90, and 70%) and washed in distilled H2O. Endogenous peroxidase activity was blocked by immersing sections in 0.3% H2O2 (Sigma, H1009) diluted in H2O for 30 min. To retrieve antigens, sections were boiled in 0.01 M citrate (pH 6.0). Sections were blocked in normal goat serum diluted 1:60 in 0.1% BSA in PBS. Sections were incubated with the primary antibody overnight at 4 °C for Caspase-4/11 (bs-6858R, Bioss). Biotinylated secondary antibody was added and detected using the rabbit peroxidase ABC kit (Vector Laboratories, PK-4001), according to the manufacturer’s instructions. Substrate was developed using the NovaRed kit (Vector Laboratories, SK-4800). Nuclei were counterstained with heamatoxylin, and sections were dehydrated through graded ethanol solutions, cleared in xylene, and mounted in di-nbutylehthalate in xylene (Thermo Scientific, LAMB-DPX). Staining was analysed with a NIKON ECLIPSE-E800 microscope, and images were captured with a Leica DFC420 camera using the LAS software (Leica). 10–15 random images were captured per section and the percentage of positively stained cells determined from the total number of cells before an average per mouse was calculated.

### Telomere-associated DNA damage response (DDR) foci (TAF) assay in mouse tissue samples

Determination of TAFs was performed by Formalin-Fixed Paraffin-Embedded immuno-Fluorescence in-sity hybridization (FFPE immune-FISH). FFPE tissue sections were deparaffinized in Histoclear, hydrated in ethanol series for 5 min each and washed 2×5 min in distilled water. Antigen retrieval was performed using 0.01 M citrate buffer (pH 6.0) and heated until boiling for 10 min. Sections were cooled down followed by two washes in distilled water for 5 min and 5 min PBS. Next, sections were blocked in normal goat serum (1:60) in BSA/PBS for 30 min and incubated with primary antibody overnight at 4 °C with rabbit monoclonal anti-γH2AX (1:200, 9718; Cell Signaling). The next day, sections were incubated with a goat anti-rabbit biotinylated secondary antibody (1:200, PK-6101; Vector Labs) for 45 min at RT. Following three PBS washes, tissues were incubated with fluorescein avidin Cy5 (1:500, SA-1500-1; Vector Labs) for another 25 min at RT. Sections were then washed 3×5 min in PBS and cross-linked by incubation in 4% paraformaldehyde in PBS for 20 min. Sections were washed in PBS 3x 5 min and then dehydrated in graded cold ethanol solutions (70, 90, 100%) for 3 min each. Tissues were then allowed to air-dry then denatured in 10 μl of hybridization mix (70% deionized formamide (Sigma), 25 mM MgCl_2_, 1 M Tris pH 7.2, 5% blocking reagent (Roche) containing 2.5 μg/ml Cy-3-labeled telomere-specific (CCCTAA) peptide nucleic acid probe (Panagene) for 10 min at 80 °C and then incubated for 2 h at RT in a dark humidified chamber to allow hybridization to occur. Sections were washed in 70% formamide in 2 × SCC for 10 min, followed by a wash in 2 × SSC for 10 min, and a PBS wash for 10 min. Tissues were then mounted using ProLong Gold Antifade Mountant with DAPI (Invitrogen). Sections were imaged using in-depth Z stacking (a minimum of 40 optical slices with 63× objective on a Leica Dmi8 microscope). Manual quantification was performed using FIJI (https://imagej.net/software/fiji/).

### Broad-band autofluorescence (lipofuscin accumulation) analysis

Broad-band autofluorescence was acquired from sections cut at 3 μm using X20 objective (Leica DM550B). Sections were excited at 458 nm and fluorescence emission captured above 475 nm. Fluorescence intensity per airway epithelium was quantified using ImageJ software and divided by background emission. At least 10 small airways per mouse was analysed.

### Data plotting and statistical analysis

Data plotting and statistical analysis were performed using Prism 7 (Graph Pad). Statistical significance for each experiment was established by two-tailed unpaired or paired *t* test, Mann–Whitney *U* test and one-way or two-way ANOVA, as appropriate, using the built-in analysis tools of Prism 7. Statistical tests, chosen based on the nature of the comparison being made and the standard tests used in the field, are indicated in the figure legends. Underlying assumptions for these tests, including sample independence, variance equality, and normality were assumed to be met although not explicitly examined. One-way ANOVA was followed by Tukey’s multiple comparison test. All measurements were taken from distinct samples, as noted in figure legends, and no data were excluded. Sample sizes were based in standard protocols in the field. Unless otherwise stated, at least three biological independent replicates were performed for each experiment. Asterisks denote *p* value as follows: *p < 0.05, **p < 0.01, ***p < 0.001, ****p < 0.0001.

## Supplementary information

supplemental legends and tables

Supplementary figures

Dataset 1

Reproducibility checklist

## Data Availability

The RNA-seq transcriptomic data in this study are available at the Gene Expression Omnibus (GEO) database with number GSE188373. The rest of the data supporting the present study are available from the corresponding author upon reasonable request.
