# Supplementary material for: Cytoplasmic innate immune sensing by the caspase-4 non-canonical inflammasome promotes cellular senescence
Source: Cell Death Differ. 2021 Dec 16;29(6):1267–82. doi: 10.1038/s41418-021-00917-6 (PMC9177556; doi:10.1038/s41418-021-00917-6)
Supplement: Supplementary file 1 — supplemental legends and tables [file 41418_2021_917_MOESM1_ESM.pdf]

Title

Cytoplasmic innate immune sensing by the caspase-4 non-canonical inflammasome promotes cellular senescence.

Authors

Irene Fernández-Duran[1], Andrea Quintanilla[1], Núria Tarrats[1], Jodie Birch[2], Priya Hari[1], Fraser R. Millar[1], Anthony B. Lagnado[3], Vanessa Smer-Barreto[1], Morwenna Muir[1], Valerie G. Brunton[1], João F. Passos[3], Juan Carlos Acosta[1,4] *

Affiliations

[1]Cancer Research UK Edinburgh Centre, MRC Institute of Genetics and Cancer, University of Edinburgh, Crewe Road, Edinburgh EH4 2XR, UK.

[2]MRC London Institute of Medical Sciences, Hammersmith Hospital Campus, Du Cane Road, London, W12 0NN, UK.

[3]Robert and Arlene Kogod Center on Aging, Mayo Clinic, Rochester, MN, 55905 USA.

[4]Instituto de Biomedicina y Biotecnología de Cantabria, IBBTEC (CSIC, Universidad de Cantabria). C/ Albert Einstein 22, Santander, 39011, Spain.

*Correspondence to: juan-carlos.acosta@ed.ac.uk, or acostajc@unican.es

Supplementary Information

Supplementary Fig. S1 LPS-mediated caspase-4 activation induces a senescent phenotype in human primary fibroblasts – related to Figure 1.

(**A**) IMR90 fibroblasts were un-transfected (UnT), mock-transfected (mock), transfected with MDP (1 µg) or increasing concentrations of LPS (0.1 or 1 µg LPS). Cell viability was measured 2 h after transfection. Graph bars, error bars and dots represent respectively the mean ± s.e.m. and the individual values of 3 independent experiments. Statistical analysis was performed using one-way analysis of variance (ANOVA).

(**B**) Representative images of IMR90 cells mock-transfected (left) or transfected with 0.1 µg LPS (middle) or 1 µg LPS (right) under brightfield microscopy 24 h after transfection.

(**C**) IMR90 or 293T cells were un-transfected (mock) or transfected with 1 µg LPS cells (LPS-T). To confirm that pyroptosis is dependent on intracellular localization of LPS, IMR90 were also treated with 1 µg LPS cells without further transfection (LPS - UnT). Cell viability was measured 2.5 h after transfection. Data from a single representative experiment.

(**D**) mRNA expression analysis of the inflammatory caspases *CASP1*, *CASP4* and *CASP5* in IMR90 and THP1 cells by RT-qPCR as indicated. (*n.d.)*: transcripts not detected.

(**E**) Cells were treated as shown in Figure 1A. *CASP1* and *CASP4* expression were targeted by shRNA before LPS transfection and *CASP1* mRNA relative expression was quantified by RT-qPCR 48 h after LPS transfection. Graph bars, error bars and dots represent respectively the mean ± s.e.m. and the individual values of 3 independent experiments. Statistical analysis was performed using one-way analysis of variance (ANOVA).

(**F**) Cells were treated as shown in Figure 1A. *CASP1* and *CASP4* expression were targeted by shRNA before LPS transfection and protein expression analysis for Caspase-1, Caspase-4 and β-Actin as loading control was performed by western blot.

(**G**) Cells were treated as shown in Figure 1A. *CASP1* and *CASP4* expression were targeted by shRNA before LPS transfection and cell viability was measured 24 h after LPS transfection. Graph bars, error bars and dots represent respectively the mean ± s.e.m. and the individual values of 3 independent experiments. Statistical analysis was performed using one-way analysis of variance (ANOVA).

(**H**) Related to Figure 1 G-J. IMR90 cells were infected with an empty pGIPZ vector (vector) or shRNA targeting either *CASP4* (shC4), *GSDMD* (shGSDMD) or *TP53* (shP53) and mRNA expression analysis for *CASP4*, *GSDMD* and *TP53* was performed as indicated. Graph bars, error bars and dots represent respectively the mean ± s.e.m. and the individual values of 3 independent experiments. Statistical analysis was performed using one-way analysis of variance (ANOVA).

(**I**) Related to Figure 1 G-J. IMR90 cells were infected with an empty pGIPZ vector (vector) or shRNA targeting either *CASP4* (shC4), *GSDMD* (shGSDMD) or *TP53* (shP53) before transfection with 0.1 µg LPS and cell viability was measured 24 h after LPS transfection. Graph bars, error bars and dots

represent respectively the mean ± s.e.m. and the individual values of 3 independent experiments. Statistical analysis was performed using two-tailed Student's *t*-test.

(**J**) Cell viability time-course following LPS transfection. IMR90 fibroblasts were mock-transfected, transfected with 1 µg MDP, or with increasing concentrations of LPS (0.01, 0.1 or 1 µg LPS). Cell viability was measured 5, 24, 48 and 72 h after transfection. Graph bars, error bars and dots represent respectively the mean ± s.e.m. and the individual values of 3 independent experiments. Statistical analysis was performed using two-way analysis of variance (ANOVA).

(**K**) IMR90 fibroblasts were mock-transfected or transfected with increasing concentrations of LPS (0.1 or 1 µg LPS). Cell viability was measured 2.5 h after transfection and viable cells were replated and cultured for further 48 h before measuring cell viability again. Bars show a single representative experiment.

(**L-M**) IMR90 fibroblasts were mock-transfected (control), transfected with 1 µg MDP, or with increasing concentrations of LPS (0.01, 0.1 or 1 µg LPS) and the acquisition of senescent features after LPS transfection was assessed by immunofluorescence of senescence markers and SA-β-Galactosidase activity. BrdU incorporation and p16^{INK4a}, p21^{CIP1} and caspase-4 protein levels were measured by immunofluorescence 48 h after transfection. SA-β-Galactosidase activity was determined 4 days after transfection. Representative pictures (left) of immunofluorescence staining for p16^{INK4a}, p21^{CIP1} and caspase-4 of mock-transfected (control) and cells transfected with LPS (0.01 LPS) are shown. Graph bars, error bars and dots represent respectively the mean ± s.e.m. and the individual values of 3 independent experiments. Statistical analysis was performed using one-way analysis of variance (ANOVA).

*****P* < 0.0001, ****P* < 0.001, ***P* < 0.01, and **P* < 0.05. ns, not significant. Scale bar = 0.1 mm and 250 µm as indicated.

Supplementary Fig. S2 LPS-induced senescence is not a consequence of pyroptotic cell death, and can be induced in cells of distinct epithelial origin – related to Figure 2.

(A) IMR90 cells were transfected with 5 ug/mL of LPS, MDP or without PAMP (MOCK) as indicated, and cell culture viability was measured 24 hours after transfection. Graph bars, error bars and dots represent respectively the mean ± s.e.m. and the individual values of 3 independent experiments. Statistical analysis was performed using one-way analysis of variance (ANOVA).

(B) Cell culture supernatants from cells in (**A**) were transferred to healthy IMR90 (CM transfer). As an experimental control, cells treated with Etoposide, and cells transfected with 5 ug/ml LPS and MDP, or MOCK transfected (transfected) were seeded at same density as healthy IMR90 cells one day prior to the supernatant transfer. Representative images of crystal violet-stained cultured cells 14 days (upper panels), and of SA-β-Galactosidase-stained cells 5 days (lower panels) after treatment are shown.

(C) Relative cell content was quantified from the crystal violet experiment described in (**B**). Graph bars, error bars and dots represent respectively the mean ± s.e.m. and the individual values of 3 independent experiments. Statistical analysis was performed using one-way analysis of variance (ANOVA).

(**D**) Graph representing the proportion (%) of cells positive for SA-β-Galactosidase activity determined after treatments described in (**B**). Graph bars, error bars and dots represent respectively the mean ± s.e.m. and the individual values of 3 independent experiments. Statistical analysis was performed using one-way analysis of variance (ANOVA).

(**E**) CAPAN-1, PSN-1, A549 and HCT116 cells were transfected with 1 or 10 ug/mL of LPS, 10 ug/mL of MDP or without PAMP (MOCK) as indicated, and viable cells were seeded at same density 24 hours after transfection and staining with crystal violet to assess cell proliferation. Representative images of crystal violet-stained cultured cells 14 days.

(**F-G**) CAPAN-1, PSN-1, A549 and HCT116 cells were transfected with 1 or 10 ug/mL of LPS, 10 ug/mL of MDP or without PAMP (MOCK) as indicated, and viable cells were seeded at same density 24 hours after transfection, cultured for additional 5 days and tested for SA-β-Galactosidase activity. Representative images (**F**) and graph representing the proportion (%) of cells positive for SA-β-Galactosidase activity determined after treatments described in (**G**) are shown. Graph bars, error bars and dots represent respectively the mean ± s.e.m. and the individual values of 3 independent experiments. Statistical analysis was performed using one-way analysis of variance (ANOVA).

(**H-J**) *CASP4*, *CASP1* or *RAS*^{G12V} were overexpressed in IMR90 cells and (**H**) protein amounts of CASP4 and CASP1 were analyzed by immunoblotting. Expression of β-Actin was assessed as a loading control. (**I**) BrdU incorporation and SA-β-Galactosidase activity were determined 4 days after seeding equal number of cells. (**J**) Relative cell content (left) was quantified 15 days after equal number of cells were seeded; representative images (right) of crystal-violet stained cells are shown. Graph bars, error bars and dots represent respectively the mean ± s.e.m. and the individual values of 3 independent experiments. Statistical analysis was performed using one-way analysis of variance (ANOVA).

(**K**) *CASP4* was overexpressed in IMR90 cells and cells were transfected with LPS (0.1 µg LPS). Caspase-4 and p16^{INK4a} protein levels were analyzed by immunoblotting 48 h after LPS transfection. Expression of β-Actin was assessed as a loading control.

(**L-M**) Related to Figure 1 G-J. IMR90 cells were infected with an empty pGIPZ vector (vector) or shRNA targeting either *CASP4* (shC4), *GSDMD* (shGSDMD) or *TP53* (shP53) before transfection with 0.1 µg LPS. p53 (**L**) and caspase-4 (**M**) protein levels were measured by immunofluorescence 48 h after LPS transfection. Graph bars, error bars and dots represent respectively the mean ± s.e.m. and the individual values of 3 independent experiments. Statistical analysis was performed using one-way analysis of variance (ANOVA).

Supplementary Fig. S3 LPS-mediated caspase-4 induced senescence is independent on inflammasome priming and its catalytical function – related to Figure 2 and 3.

(**A**) IMR90 cells were treated with MDP (1 µg/mL), LPS, (1 µg/mL) Pam2CSk4 (0.05 µg/mL) or Pam3CSk4 (0.5 µg/mL). To analyze inflammasome priming, *IL1B* mRNA relative expression was quantified by RT-qPCR 3, 6, 24 and 48 h after the addition of ligands. Data from a single representative experiment.

(**B**) IMR90 cells infected with toll-like receptor-2 (TLR2) expressing vector or control empty vector (EV) were primed with 10 µg/ml of A-SAA for 3 hours prior to electroporation with LPS (0.1 µg) to activate caspase-4. *IL1B* mRNA relative expression was quantified by RT-qPCR 48 hours after LPS transfection. Graph bars, error bars and dots represent respectively the mean ± s.e.m. and the individual values of 3 independent experiments. Statistical analysis was performed using one-way analysis of variance (ANOVA).

(**C**) Samples from (**B**) were analyzed for *IL1A*, *IL6*, *IL8* mRNA relative expression by RT-qPCR 48 hours after LPS transfection. Graph bars, error bars and dots represent respectively the mean ± s.e.m. and the individual values of 3 independent experiments. Statistical analysis was performed using one-way analysis of variance (ANOVA).

(**D**) SA-β-Galactosidase activity staining was conducted 48 hours after treatment as in (**B**). Values represent the mean ± s.e.m. of 3 independent experiments.

(**E**) Cell proliferation capacity in experiment (**B**) was measured by BrdU incorporation assay. Graph bars, error bars and dots represent respectively the mean ± s.e.m. and the individual values of 3

independent experiments. Statistical analysis was performed using one-way analysis of variance (ANOVA).

(**F**) Analysis of *IL1B* mRNA expression by RT-qPCR in IMR90 cells infected with TLR2 expressing vector or control empty vector (EV), primed with 1 µg/mL Pam2CSK4 for 3 hours, followed by electroporation with 0.1 µg LPS for 48 hours. Graph bars, error bars and dots represent respectively the mean ± s.e.m. and the individual values of 3 independent experiments. Statistical analysis was performed using one-way analysis of variance (ANOVA).

****P < 0.0001, ***P < 0.001, **P < 0.01, and *P < 0.05. ns, not significant. Scale bar = 0.1 mm as indicted.

(**G-H**) IMR90 cells were infected with wild-type (WT) *CASP4*, catalytically inactive (C258A) *CASP4* or the empty vector (vector) prior to transfection with 1 µg LPS. (**G**) Cells in culture under brightfield microscopy 24 h after LPS transfection, at the time of viability assessment. Related to Figure 3D. (**H**) Representative images of SA-β-Galactosidase activity-stained cells 4 days after LPS transfection. Related to Figure 3F.

Scale bar = 1mm and 0.1 mm as indicated.

Supplementary Fig. S4 The caspase-4 non-canonical inflammasome is activated in Oncogene-induced senescence – related to Figure 4

(**A**) *CDKN1A* ($p21^{CIP1}$), *CDKN2A* ($p16^{INK4a}$) and *CDKN2B* ($p15^{INK4b}$) relative expression levels were measured by RT-qPCR in IMR90 cells undergoing RAS^{G12V}-OIS. Graph bars, error bars and dots represent respectively the mean ± s.e.m. and the individual values of 3 independent experiments. Statistical analysis was performed using two-tailed Student's *t*-test.

(**B**) IMR90 ER:STOP and ER:RAS cells were treated with the indicated siRNAs and 5 days after the addition of 4OHT caspase-4 protein was stained for immunofluorescence analysis. Representative images of caspase-4 stained cells are shown.

(**C**) Caspase-4 protein levels after the induction of paracrine senescence were analyzed. After 8 days of 4OHT treatment, conditioned media (CM) from IMR90 ER:STOP and ER:RAS cells was collected and added to IMR90 cells. After 48 h, BrdU incorporation (middle) and caspase-4 levels (right) were measured by immunofluorescence. Graph bars, error bars and dots represent respectively the mean ±

s.e.m. and the individual values of 3 independent experiments. Statistical analysis was performed using two-tailed Student's *t*-test.

(**D**) IMR90 cells were treated with 10 μM etoposide and 48 hour later BrdU incorporation (left) and caspase-4 (right) levels were measured by immunofluorescence. Data relative to BrdU incorporation belongs to a single representative experiment. Graph bars, error bars and dots relative to caspase-4 protein levels represent respectively the mean ± s.e.m. and the individual values of 3 independent experiments. Statistical analysis was performed using two-tailed Student's *t*-test.

(**E**) Caspase-4 oligomerization was analyzed in a RAS^{G12V}-OIS time-course. IMR90 ER:STOP and ER:RAS cells were treated with 4OHT for the indicated time, cells were harvested and subjected to DSS-crosslinking. After SDS-PAGE separation, both DSS-crosslinked samples and inputs were probed for caspase-4 following western blot procedures.

****P* < 0.001, ***P* < 0.01, and **P* < 0.05. ns, not significant. Scale bar = 250 μm as indicated.

Supplementary Fig. S5 Caspase-4 activation controls the proinflammatory SASP – related to Figure 5

(**A**) *CASP4* mRNA relative expression levels were quantified by RT-qPCR after 5 days (left) and 8 days (right) of 4OHT treatment in ER:STOP and ER:RAS cells transfected with the indicated siRNAs. Graph bars, error bars and dots represent respectively the mean ± s.e.m. and the individual values of 3 independent experiments. Statistical analysis was performed using one-way analysis of variance (ANOVA).

(**B**) Related to Figure 5A. Top five differentially expressed genes upon *CASP4*-targeting in RAS^{G12V}-OIS identified by DEG analysis 5 and 8 days after 4OHT treatment.

(**C**) Principal component analysis (PCA) of variance stabilized transformed data using a parametric fit for the dispersion. Each dot corresponds to a sample replicate.

(**D**) Heatmap and hierarchical clustering of the 30 genes with highest variance across all samples based on the total transformed data.

(**E**) Related to Figure 5B. Enrichment plots of the signature "INFLAMMATORY RESPONSE" upon *CASP4*-targeting in RAS^{G12V}-OIS 5 (top) and 8 (bottom) days after 4OHT treatment are shown. Normalized enrichment scores (NES), p-values and FDR q-values as obtained by GSEA are also shown.

(**F**) IMR90 ER:STOP or ER:RAS cells were transfected with control (NTP), *CASP1* or *CASP4*-targeting siRNA and treated with 4OHT during 8 days. IL-1α, L-1β, IL-6 and IL-8 levels were analyzed by immunofluorescence 8 days after the addition of 4OHT. Representative images as used for the high content analysis are also shown. Graph bars, error bars and dots represent respectively the mean ± s.e.m. and the individual values of 3 independent experiments. Statistical analysis was performed using one-way analysis of variance (ANOVA).

(**G**) IMR90 ER:STOP and ER:RAS cells were transfected with control (NTP), two individual *CASP4*-targeting siRNAs (CASP4-1 and CASP4-2) or a pool of 4 different siRNA sequences targeting *CASP4* (CASP4-p), and treated with 4OHT or not as indicated. *CASP4* mRNA relative expression was quantified by RT-qPCR in IMR90 cells 5 days after the addition of 4OHT. Caspase-4 and IL1-β protein expression were analyzed by immunoblotting. Expression of β-Actin was assessed as a loading control. Graph bars, error bars and dots represent respectively the mean ± s.e.m. and the individual values of 3 independent experiments. Statistical analysis was performed using two-tailed Student's *t*-test.

(**H**) To examine the role of caspase-4 on the SASP during paracrine senescence, IMR90 cells were treated with conditioned media (CM) from IMR90 ER:STOP and ER:RAS cells after 8 days of 4OHT treatment, and concomitantly transfected with control (NTP), *CASP1* or *CASP4*-targeting siRNA. After 48 h, *IL1A*, *IL1B*, *IL8*, *IL6*, *CASP1* and *CASP4* mRNA relative expression levels were measured by RT-qPCR. Graph bars, error bars and dots represent respectively the mean ± s.e.m. and the individual values of 3 independent experiments. Statistical analysis was performed using two-tailed Student's *t*-test. ****$P < 0.0001$, ***$P < 0.001$, **$P < 0.01$, *$P < 0.05$ and ns, not significant. Scale bar = 250 μm as indicated.

Supplementary Fig. S6 Caspase-4 contributes to the arrest in cell proliferation **in OIS – related to Figure 6**

(**A**) Time-course of GSDMD mRNA expression during *RAS*^{G12V}-OIS. *GSDMD* mRNA relative expression was quantified by RT-qPCR in IMR90 ER:STOP and ER:RAS cells 0, 2, 4, 6 and 8 days after 4OHT addition. Graph lines and dots represent respectively the mean and the individual values of 3 independent experiments. Statistical analysis was performed using two-tailed Student's *t*-test.

(**B**) IMR90 ER:STOP and ER:RAS cells were treated with 4OHT during 4 or 8 days as indicated. Caspase-4, full-length (FL) and N-terminal (NT) Gasdermin-D, IL-1β and IL-8 levels were analyzed by immunoblotting. Expression of β-Actin was assessed as a loading control.

(**C**) Oncogenic H-Ras^{G12V} was overexpressed in IMR90 cells to induce OIS. Gasdermin-D expression was analysed by immunoblotting in control cells infected with an empty vector (MSCV) and senescent cells. Expression of β-Actin was assessed as a loading control.

(**D**) IMR90 cells expressing ER-STOP and ER-RAS were induced to senescence with 4OHT during 8 days and trypan blue exclusion assay showing cell viability with the indicated cultures. As a positive control of cell death, IMR90 ER-RAS cells were treated 0.1 μM Ouabain 6 days after 4OHT induction. Graph bars, error bars and dots represent respectively the mean ± s.e.m. and the individual values of 3 independent experimental replicates. Statistical analysis was performed using one-way analysis of variance (ANOVA).

(**E**) IMR90 ER:STOP and ER:RAS cells were transfected with control, *CASP1*, *CASP4* or *GSDMD*-targeting siRNAs. To analyze knockdown efficiency, *CASP1*, *CASP4* and *GSDMD* mRNA relative expression levels were quantified by RT-qPCR after 5 days of 4OHT treatment. Graph bars, error bars and dots represent respectively the mean ± s.e.m. and the individual values of 3 independent experiments. Statistical analysis was performed using one-way analysis of variance (ANOVA).

(**F**) IMR90 ER:STOP and ER:RAS cells were transfected with control, *CASP1*, *CASP4* or *GSDMD*-targeting siRNAs. *IL1A*, *IL1B* and *IL8* mRNA relative expression levels were quantified by RT-qPCR after 5 days of 4OHT treatment. Graph bars, error bars and dots represent respectively the mean ± s.e.m. and the individual values of 3 independent experiments. Statistical analysis was performed using one-way analysis of variance (ANOVA).

(**G**) IMR90 ER:STOP and ER:RAS cells were transfected with the indicated siRNAs and secreted IL-1β was quantified by ELISA 6, 7, and 8 days after 4OHT addition. Statistical analysis was performed using one-way analysis of variance (ANOVA) comparing control senescent cells (ER:RAS siNTP) to the other conditions 8 days after 4OHT addition. Graph lines and dots represent respectively the mean and the individual values of 3 independent experiments.

(**H-I**) IMR90 ER:STOP and ER:RAS cells were infected with wild-type (WT) *CASP4*, catalytically inactive (C258A) *CASP4* or the empty vector (vector). Caspase-4 and IL-1β levels were analyzed by

immunoblotting (**H**) and *IL1A* and *IL1B* mRNA relative expression levels were measured by RT-qPCR (**I**) 4 days after the addition of 4OHT.

(**J**) IMR90 ER:STOP and ER:RAS cells were transfected with control or *CASP4*-targeting siRNAs and SA-β-Galactosidase activity was determined 8 days after the addition of 4OHT (left). Representative images for SA-β-Galactosidase activity are shown (right). Graph bars, error bars and dots represent respectively the mean ± s.e.m. and the individual values of 3 independent experiments. Statistical analysis was performed using two-tailed Student's *t*-test.

(**K**) Related to Figure 5B. Enrichment plots of the signatures "G2M CHECKPOINT" (left) and "E2F TARGETS" (right) upon *CASP4*-targeting in *RAS*^{G12V}-OIS IMR90 cells 5 days after 4OHT treatment.

(**L**) IMR90 ER:STOP and ER:RAS cells were transfected with control (NTP), *CASP1* or *CASP4*-targeting siRNAs. After 5 days of 4OHT treatment, caspase-4 proIL-1β and pRb were analyzed by immunoblotting. Expression of β-Actin was assessed as a loading control.

(**M**) IMR90 cells were infected with an empty pGIPZ vector (vector) or shRNA targeting either *CASP1* (shC1) or *CASP4* (shC4) before transfection with 0.1 µg LPS. *CCNA1*, *CDC6* and *BUB1* mRNA relative expression were quantified by RT-qPCR 48 h after LPS transfection. Graph bars, error bars and dots represent respectively the mean ± s.e.m. and the individual values of 3 independent experiments. Statistical analysis was performed using one-way analysis of variance (ANOVA).

(**N-O**) IMR90 ER:STOP and ER:RAS cells were transfected with control, *CASP1*, *CASP4* or *GSDMD*-targeting siRNAs. (**N**) Caspase-4, Gasdermin-D and IL1-β protein expression was investigated by immunoblotting as indicated, using β-Actin was expression as a loading control, and (**O**) BrdU incorporation was measured by immunofluorescence 5 days after 4OHT addition. Graph bars, error bars and dots represent respectively the mean ± s.e.m. and the individual values of 5 independent experiments. Statistical analysis was performed using two-tailed Student's *t*-test.

*****P* < 0.0001, ****P* < 0.001, ***P* < 0.01, and **P* < 0.05. ns, not significant. Scale bar = 0.1 mm as indicated.

Supplementary Fig, S7 The role of the caspase-4 non-canonical inflammasome in cellular senescence in vivo:

(**A**) Evaluation of Caspase-11 antibody specificity for immunohistochemistry. Mouse embryonic fibroblasts (MEFs) where transfected with siRNA smartpools against CASP11 or non-targeting controls

and cultured for 24 hours, pelleted, fixed in formalin, embedded in paraffin, and processed like murine tissue for histology. Images are representative images of immunostaining using caspase-11 or secondary antibodies only to compare background levels.

(**B**) Model representing the uncoupled mechanisms by caspase-4 regulating the arrest in cell proliferation by tumour suppressor activation (p53-p21^{CIP-1} and p16^{INK4A}) and promoting the SASP upstream of caspase-1 and IL-1β priming by TLR2 in cellular senescence.

Table S1: List of antibodies used in this study

Antibody	Source	Reference
Anti-Caspase-4	Santa Cruz	4B9
Anti-BrdU	BD Biosciences	555627
Anti-IL-1α	R&D	MAB200
Anti-IL-1β	R&D	MAB201
Anti-IL-6	R&D	AF206NA
Anti-IL-8	R&D	MAB208
Anti-p21	Sigma	P1484
Anti-p16 (IF)	ProteinTech	10883-1-AP
Mouse-Alexa Fluor 488	ThermoFisher	A-11029
Rabbit-Alexa Fluor 594	ThermoFisher	A-11037
Goat-Alexa Fluor 594	ThermoFisher	A-11058
Anti-Caspase-1	Adipogen	Bally-1
Anti-Gasdermin-D	Novus Biologicals	NBP2-33422
Anti-p16 (WB)	Santa Cruz	JC-8
Anti-p53	Santa Cruz	DO-1
Anti-pRb	BD Pharmigen	554136
Anti-β-Actin	Santa Cruz	I-19
Anti-Mouse-HRP	Sigma	A2554
Anti-Rabbit-HRP	Sigma	A0545
Anti-Goat-HRP	Sigma	2020

Table S2: siRNA reagents used in this study

Target	Source	Identifier
ON-TARGETplus Non-targeting Pool	Dharmacon	D-001810-10
ON-TARGETplus CASP1-targeting Pool	Dharmacon	LQ-004401-00
ON-TARGETplus CASP4-targeting Pool	Dharmacon	LQ-004404-00
ON-TARGETplus CASP4-targeting #1	Dharmacon	J-004404-06
ON-TARGETplus CASP4-targeting #2	Dharmacon	J-004404-08
ON-TARGETplus GSDMD-targeting Pool	Dharmacon	LQ-016207-00

Table S3: Primers used for mRNA gene expression analysis

Gene	Forward	Reverse
ACTB	CATGTACGTTGCTATCCAGGC	CTCCTTAATGTCACGCACGAT
CASP4	GAGAAGCAACGTATGGCAGG	GGAATTCTTCATGAGGACAAAGC
IL1B	TGCACGCTCCGGGACTCACA	CATGGAGAACACCACTTGTTGCTCC
IL1A	AGTGCTGCTGAAGGAGATGCCTGA	CCCCTGCCAAGCACACCCAGTA
IL6	CCAGGAGCCCAGCTATGAAC	CCCAGGGAGAAGGCAACTG
IL8	GAGTGGACCACACTGCGCCA	TCCACAACCCTCTGCACCCAGT
CASP1	CAACTACAGAAGAGTTTGAGG	AACATTATCTGGTGTGGAAG
GSDMD	ATGGATGGGCAGATACAGGG	TGCTGCAGGACTTTGTGTTC
CDKN1A	CCTGTCACTGTCTTGTACCCT	GCGTTTGGAGTGGTAGAAATCT
CDKN2A	CGGTCGGAGGCCGATCCAG	GCGCCGTGGAGCAGCAGCAGCT
CDKN2B	GAATGCGCGAGGAGAACAAG	CCATCATCATGACCTGGATCG
BUB1	ACACCATTCCACAAGCTT	CGCCTGGGTACACTGTTT
CDC6	GTTCAATTCTGTGCCCGCAA	TAGCTCTCCTGCAAACATCCAG
CCNA1	CCATCGACCTCAGCAAGCA	TGGCTCCATGAGGGACACA
CCNA2	AGGAAAACTTCAGCTTGTGGG	CACAAACTCTGCTACTTCTGGG
CCNB1	TGTGTCAGGCTTTCTCTGATG	TTGGTCTGACTGCTTGCTCT
MCM6	ACTGTTCCTGGACTTCTTGG	ACGAATCAGTTCCTCTGCT
SAA1	GAGCACACCAAGGAGTGATTT	GAAGCTTCATGGTGCTCTCT
SAA2	GCTGCAGAAGTGATCAGCAAT	CAGCGAGTCCTCCGCAC
CASP5	TTGCGAAAGAATCGCGTGGCTCAT	CACCTCTGCAGGCCTGGACAATGATGAC
