# Supplementary figures and images for: Cytoplasmic innate immune sensing by the caspase-4 non-canonical inflammasome promotes cellular senescence
Source: Cell Death Differ. 2021 Dec 16;29(6):1267–82. doi: 10.1038/s41418-021-00917-6 (PMC9177556; doi:10.1038/s41418-021-00917-6)

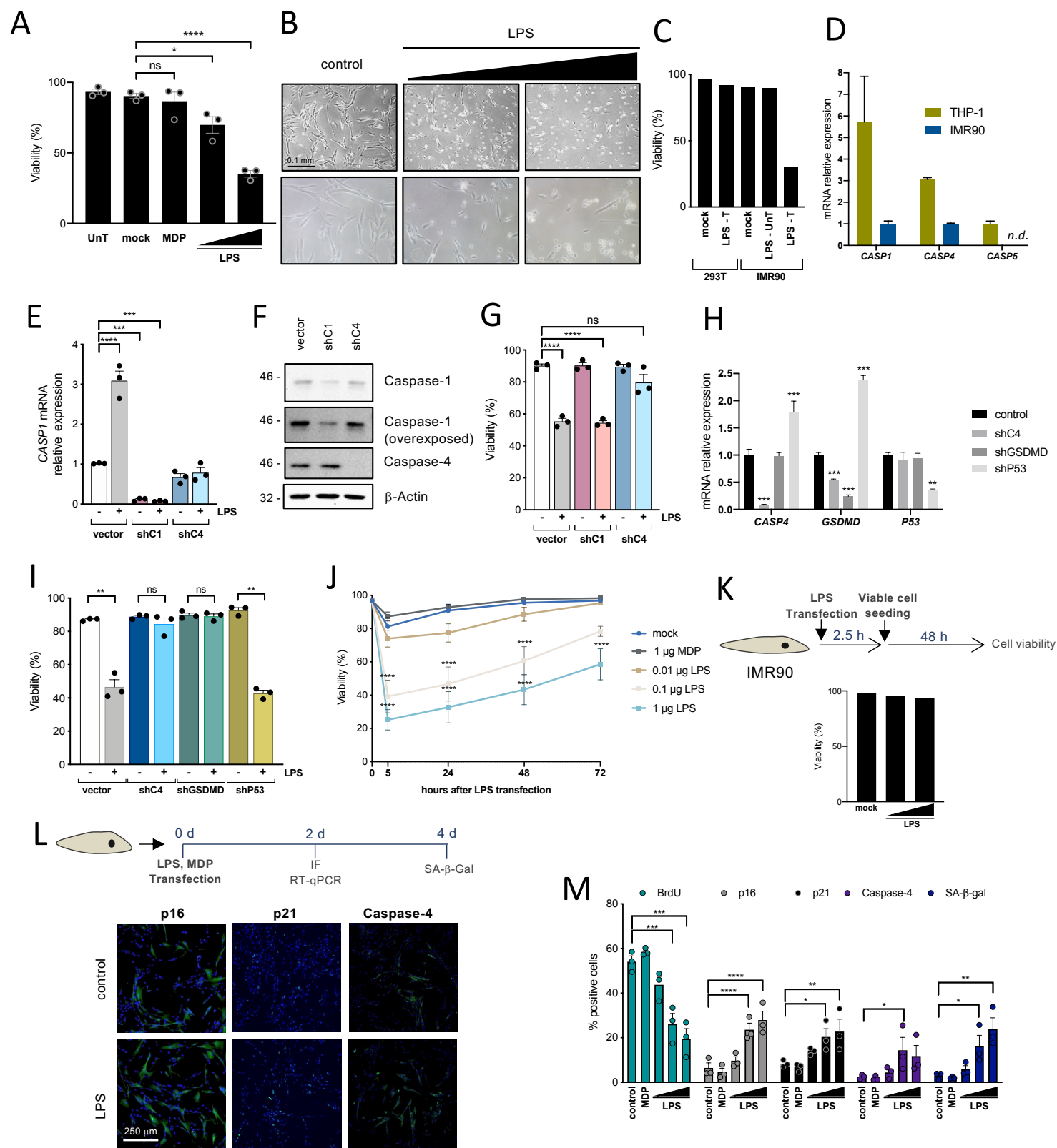

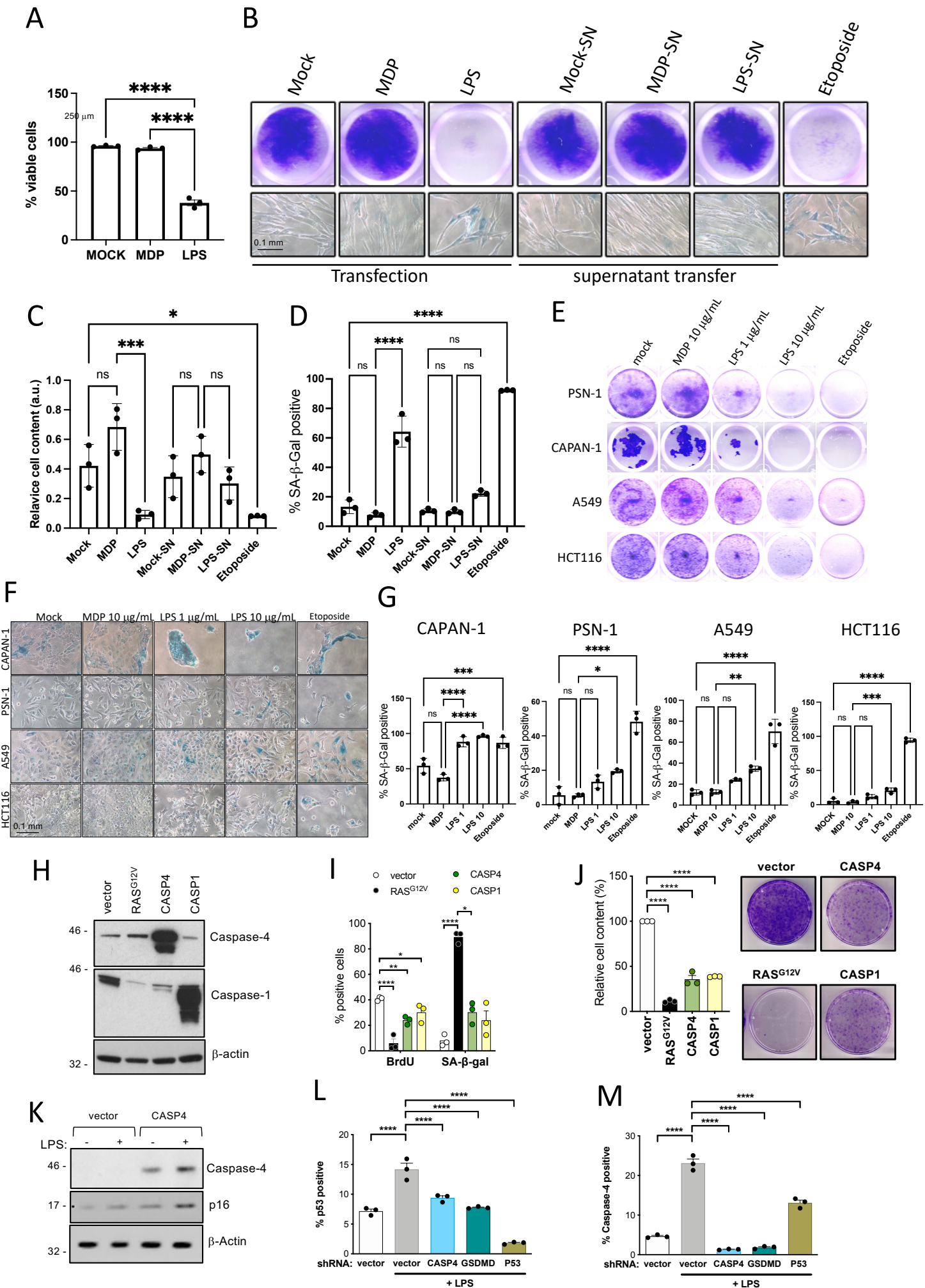

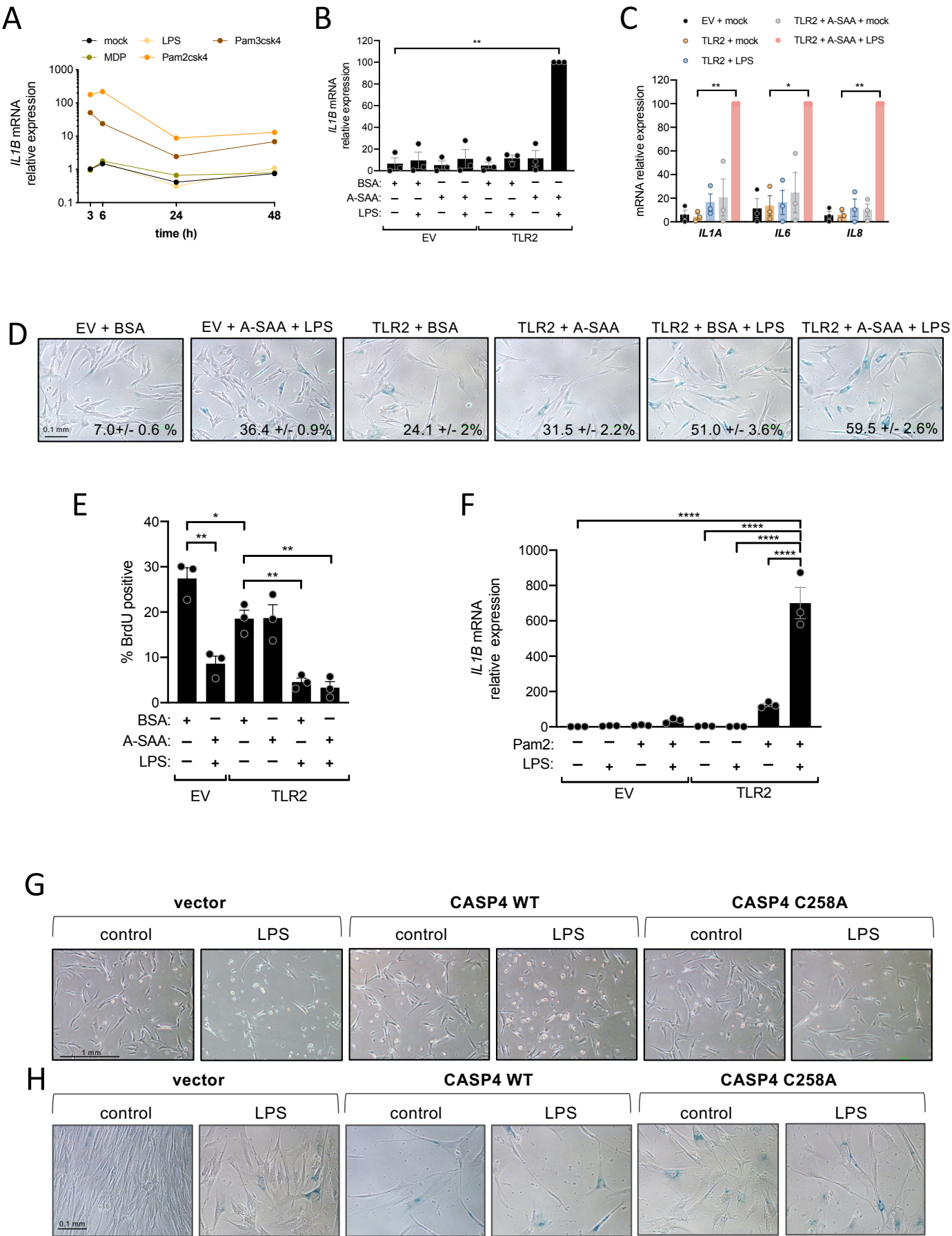

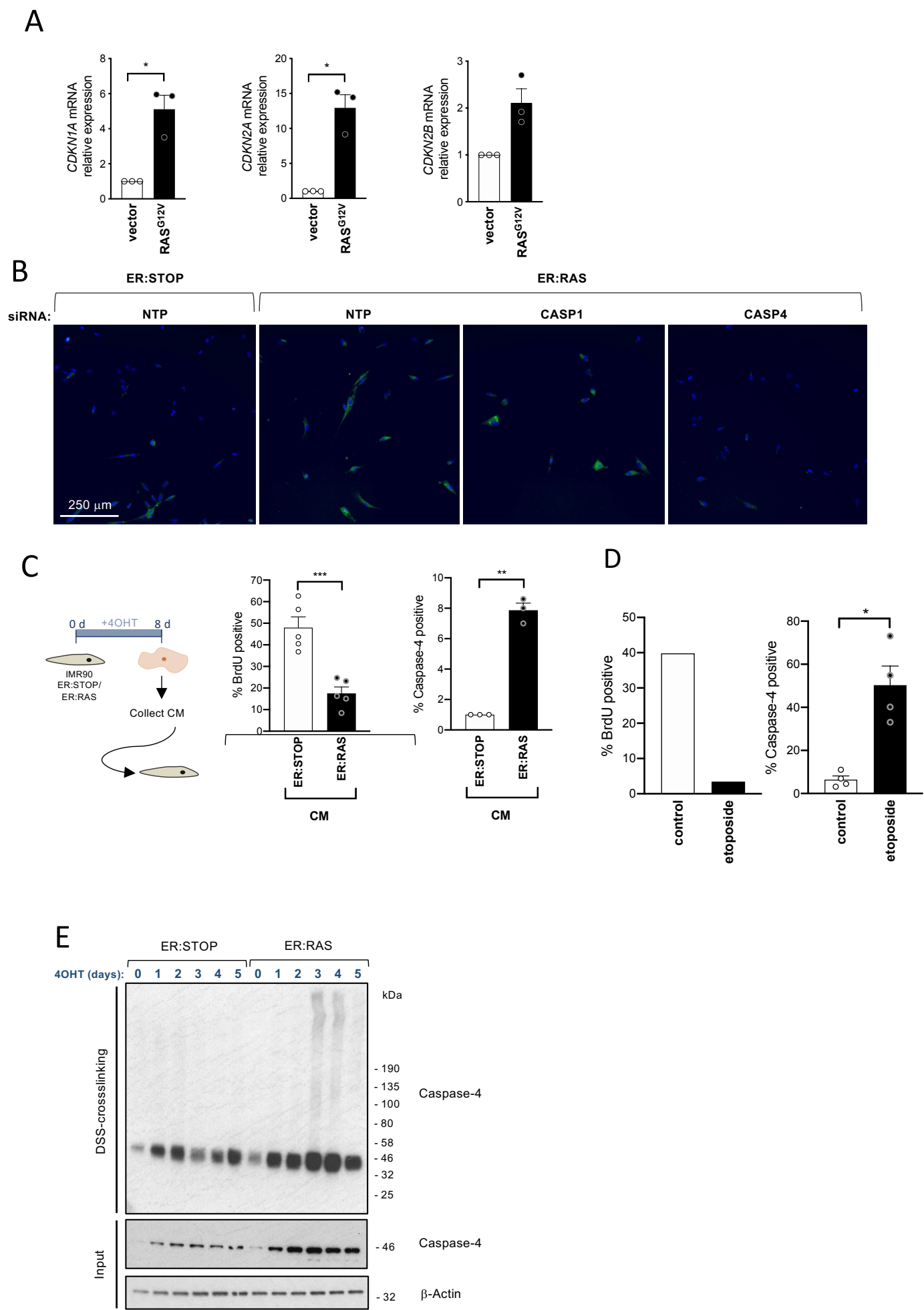

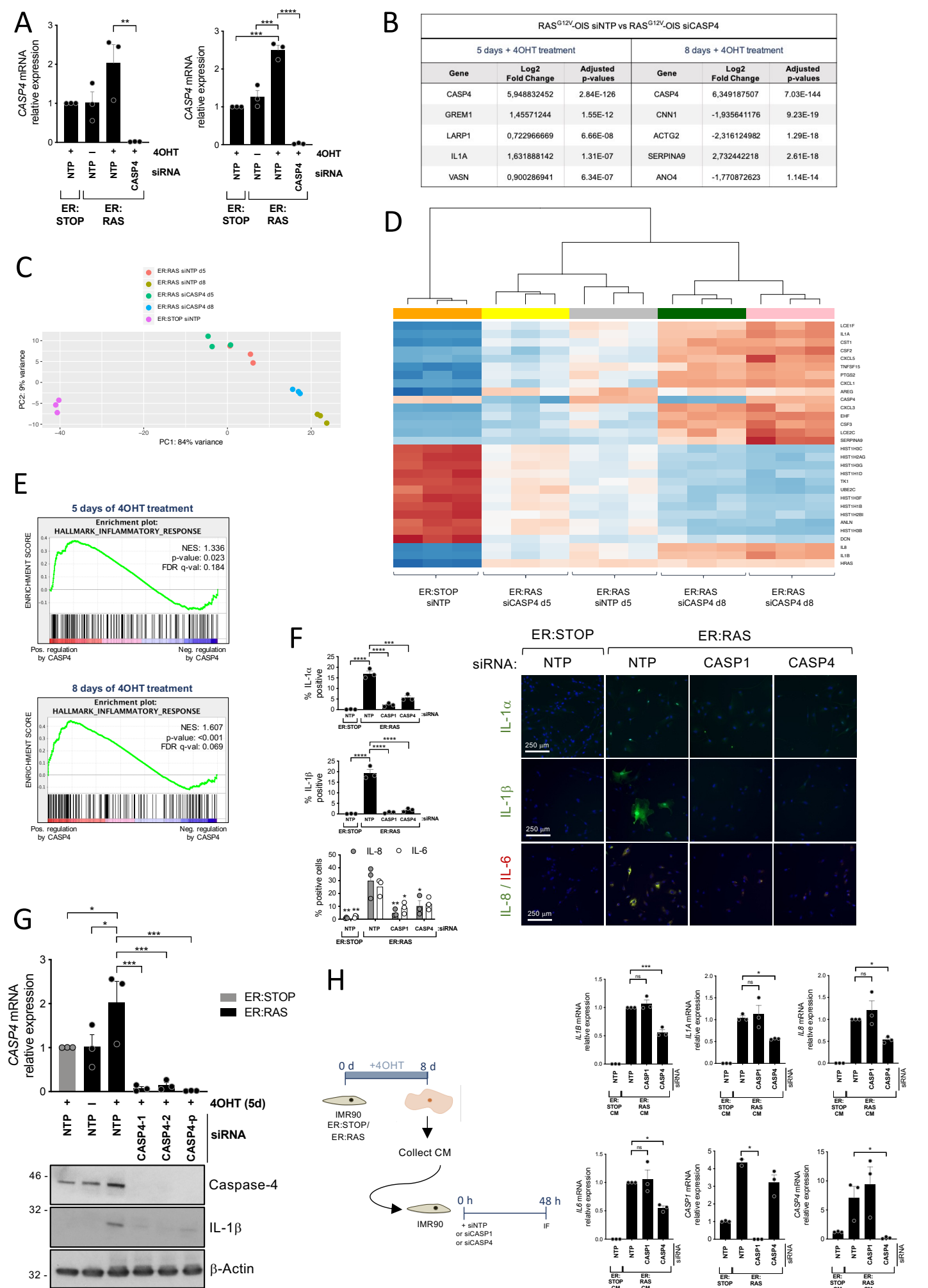

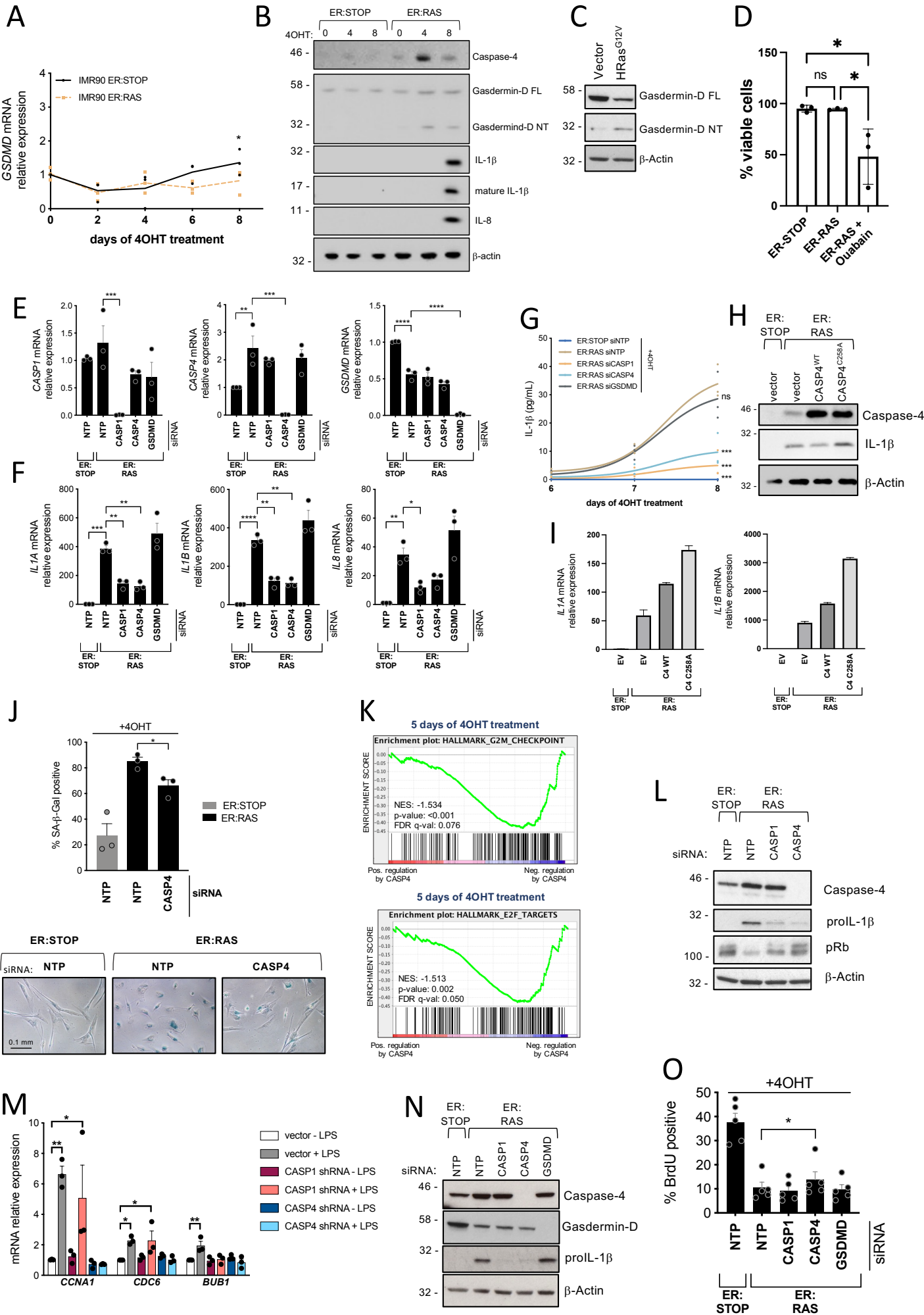

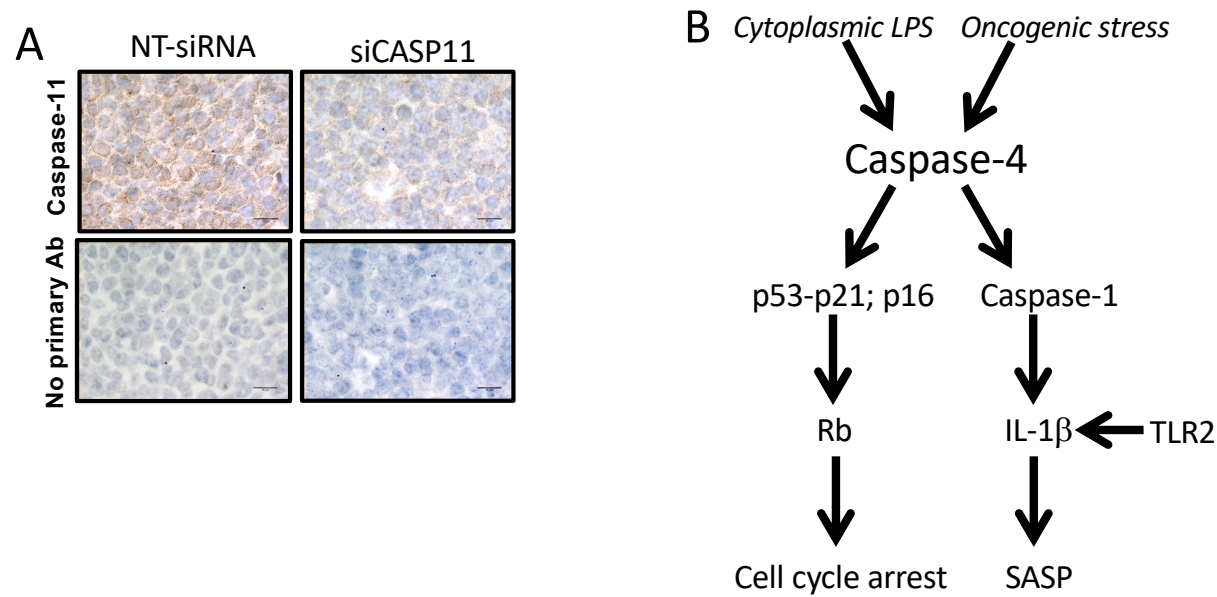

Supplement: Supplementary file 2 — Supplementary figures [file 41418_2021_917_MOESM2_ESM.pdf]
